# A Mixed-Method Approach for Quantifying Illegal Fishing and Its Impact on an Endangered Fish Species

**DOI:** 10.1371/journal.pone.0143960

**Published:** 2015-12-01

**Authors:** Christopher M. Free, Olaf P. Jensen, Bud Mendsaikhan

**Affiliations:** 1 Department of Marine and Coastal Sciences, Rutgers University, 71 Dudley Road, New Brunswick, NJ, 08901, United States of America; 2 Institute of Geoecology, Mongolian Academy of Sciences, Baruun Selbe-15, P.O. Box 81, Ulaanbaatar, 15170, Mongolia; Cornell University College of Veterinary Medicine, UNITED STATES

## Abstract

Illegal harvest is recognized as a widespread problem in natural resource management. The use of multiple methods for quantifying illegal harvest has been widely recommended yet infrequently applied. We used a mixed-method approach to evaluate the extent, character, and motivations of illegal gillnet fishing in Lake Hovsgol National Park, Mongolia and its impact on the lake’s fish populations, especially that of the endangered endemic Hovsgol grayling (*Thymallus nigrescens*). Surveys for derelict fishing gear indicate that gillnet fishing is widespread and increasing and that fishers generally use 3–4 cm mesh gillnet. Interviews with resident herders and park rangers suggest that many residents fish for subsistence during the spring grayling spawning migration and that some residents fish commercially year-round. Interviewed herders and rangers generally agree that fish population sizes are decreasing but are divided on the causes and solutions. Biological monitoring indicates that the gillnet mesh sizes used by fishers efficiently target Hovsgol grayling. Of the five species sampled in the monitoring program, only burbot (*Lota lota*) showed a significant decrease in population abundance from 2009–2013. However, grayling, burbot, and roach (*Rutilus rutilus*) all showed significant declines in average body size, suggesting a negative fishing impact. Data-poor stock assessment methods suggest that the fishing effort equivalent to each resident family fishing 50-m of gillnet 11–15 nights per year would be sufficient to overexploit the grayling population. Results from the derelict fishing gear survey and interviews suggest that this level of effort is not implausible. Overall, we demonstrate the ability for a mixed-method approach to effectively describe an illegal fishery and suggest that these methods be used to assess illegal fishing and its impacts in other protected areas.

## Introduction

Illegal, unreported, and unregulated (IUU) fishing undermine efforts to sustainably manage fish stocks and threaten fish populations worldwide [1]. Managers must know as much as possible about the extent, character (e.g., gear types, target/bycatch species, timing, location), and motivations of illegal fishing to effectively develop and implement regulations. However, quantifying illegal fishing is inherently difficult: it is generally covert and significant incentives exist for informants to withhold information [2]. Furthermore, budget and human resource constraints often restrict efforts to monitor illegal resource use, especially in developing countries [3]. There is a need to develop inexpensive yet informative methods for quantifying illegal fishing and its impacts.

Indirect observation, the use of signs of illegal activity as an indicator of non-compliance, has been commonly used to characterize illegal resource use in terrestrial systems [4], but has been infrequently used in marine systems [5], and to our knowledge, has never been used in freshwater systems. In marine systems, dynamite blast craters [6,7] and derelict fishing gear [8] have been used as indicators of illegal fishing, but have generally failed to quantitatively measure non-compliance [5]. Most successful quantifications of illegal fishing compare the amount of derelict fishing gear inside and outside reserve boundaries [9–12], but such comparisons are of little use in places without reserves or where the areas outside reserves are undesirable to fishers. The full capacity for indirect observation to reveal rich and quantitative information about illegal fishing remains unexplored.

Indirect observation offers several advantages over other approaches for assessing illegal fishing. It does not require large amounts of labor, specialized equipment, or training and can be recorded during routine enforcement patrols or biological surveys [13]. Repeated surveys can reveal spatial and temporal patterns of non-compliance [8–10,14] that can be compared to changes in fish communities to examine the effects of illegal fishing [15]. Although indirect observation generally cannot identify specific violators or motivations for non-compliance, they can contribute to a comprehensive understanding of non-compliance when combined with other methods, such as direct questioning [9–10].

In this study, we used a mixed-method approach to evaluate the extent, character, and motivations of illegal gillnet fishing in Lake Hovsgol National Park (LHNP), Mongolia and its impact on the lake’s fish populations, especially that of the endangered endemic Hovsgol grayling (*Thymallus nigrescens*). Despite the closure of the park to gillnet fishing in 1992, illegal fishing is known to persist [16–17]. We used four complementary methods to describe this fishery and evaluate its impacts: (1) surveys for derelict fishing gear, an indirect indicator of fishing activity, to evaluate how much illegal fishing is occurring, where illegal fishing is occurring, and what gear is being used; (2) interviews with herders living within the park and park rangers to validate and contextualize the results of the surveys for derelict fishing gear; (3) biological monitoring to identify fish species vulnerable to gillnet fishing and evaluate changes in population abundance potentially caused by fishing; and (4) data-poor stock assessment methods to estimate the effort required to overexploit the Hovsgol grayling population.

Overall, we demonstrate the ability for a mixed-method approach to describe an illegal gillnet fishery and suggest that these methods could be used to effectively and inexpensively assess illegal fishing and its impacts in other protected areas.

## Methods

### Study site

**Fig 1:**
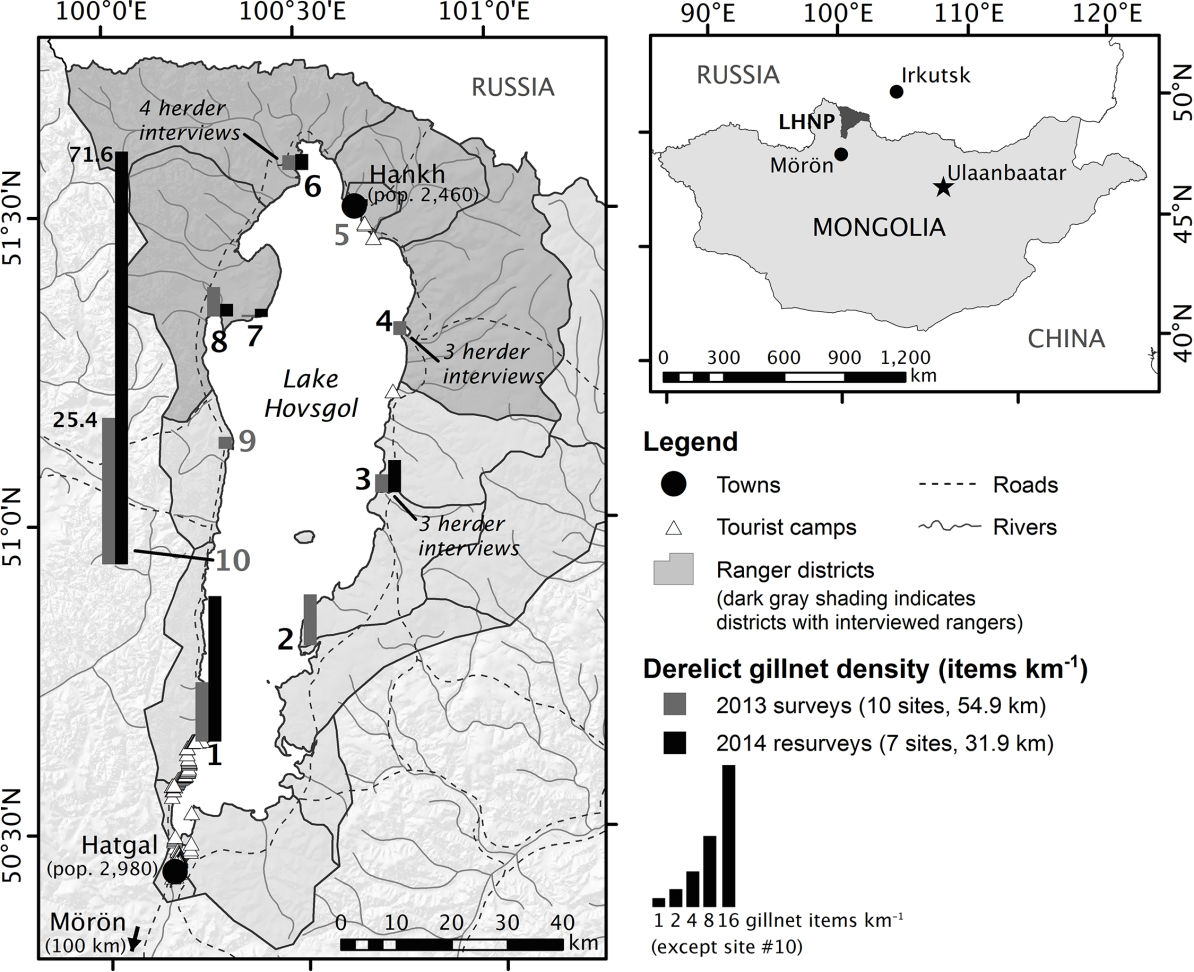
Location of shoreline surveys for derelict fishing gear, fish population monitoring sites, and interviews with park rangers and resident herders in Lake Hovsgol National Park (LHNP), Mongolia. Grey and black bars indicate the density (# km^-1^) of derelict gillnet items observed in the 2013 (n = 10) and 2014 (n = 7) surveys, respectively (note different y-axis scale for Site 10). Black site numbers indicate the seven sites where fish population monitoring surveys were conducted in 2009 and 2011–13. Solid black lines indicate the park boundary and 17 ranger districts. Five rangers from five districts (dark grey; Hankh town limits represent one district) were interviewed. Herders were interviewed at Sites 3 (n = 3), 4 (n = 3), and 6 (n = 4). Small white triangles indicate tourist camps, large black circles indicate town centers, dotted black lines indicate primitive roads, and solid gray lines indicate rivers and seasonal steams.

Lake Hovsgol (51°05’50”N, 100°30’E) is located in the mountains of northern Mongolia at the southern edge of the Siberian taiga forest. It is the 19^th^ largest lake in the world by volume (480 km^3^) and has a maximum depth of 262 m and surface area of 2,760 km^2^ [18]. The lake was established as a National Park in 1992 and is mostly undeveloped. The majority of the resident population lives in two towns on the lakeshore: Hatgal (pop. 2,980) and Hankh (pop. 2,460; [19]). Tourist camps line the southwestern shore and herding families live intermittently along the lakeshore (**Fig 1**). Most of the park’s ~35,000 annual visitors enter and remain in the southern portion of the park [20].

Lake Hovsgol has ten fish species, the most abundant of which, the Hovsgol grayling (*Thymallus nigrescens*), is endemic to the lake and is listed as endangered on the Mongolian Red List due to climate change and illegal fishing [16]. Hovsgol grayling are more common in littoral areas than pelagic areas and are most abundant along the western shore [21]. A portion of the grayling population spawns in tributary streams in late spring while another portion spawns in the littoral in late summer [22]. The prevalence, fidelity, and success of these spawning strategies are unknown.

The sparse literature on Mongolian fisheries suggests that commercial fishing for Hovsgol grayling, lenok (*Brachymystax lenok*), roach (*Rutilus rutilus*), perch (*Perca fluviatilis*), and burbot (*Lota lota*) removed as much as 200–400 tons annually before the park was established ([23]; **S1 Table**). Despite the ban on gillnet fishing, active gillnets are often observed and grayling and lenok are frequently sold in Hatgal and along the southwestern shore road. Recreational hook-and-line fishing is legal within the park and is regulated through permits and season and bag limits. Subsistence fishing during the spring spawning migration, though officially illegal, is generally tolerated.

### Surveys for derelict fishing gear

We surveyed and collected derelict fishing gear at ten sites on the Lake Hovsgol shoreline in July 2013 and resurveyed six of these sites in July 2014 (**Fig 1**). Although fishing gear found in the 2013 surveys could represent several years of accumulation and even pre-date the ban on gillnet fishing, gear found in the 2014 resurveys must represent accumulation over the preceding year, since all gear was removed from these sites during the 2013 surveys. Sites were selected as part of a long-term fish monitoring study [21]; though non-random, they provide excellent spatial coverage and access to points and bays on all sides of the lake. In 2013, we censused 54.9 km of shoreline (10 sites, 13 transects, 0.4–8.5 km each, ~13% of total shoreline) for all anthropogenic debris, including derelict fishing gear, between the water and wrack lines [24]. In 2014, we recensused 31.9 km of the original transects (7 sites/transects, 1.3–8.3 km each) for derelict fishing gear only. Because transect widths were variable, we report linear (km^-1^) rather than areal (km^-2^) debris density. Derelict fishing gear was classified into the following gillnet categories: whole net, net fragment, float line, lead line, foam float, or bottle float (**S1 Fig**); and hook-and-line categories: rod, monofilament, lure, or bobber. Bottles, string/rope, and stakes without mesh, floats, weights, or lines were not considered fishing gear. We weighed each item and measured the mesh size (knot to knot distance) of every whole gillnet or gillnet fragment.

### Interviews with herders and rangers

The Rutgers University Internal Review Board (IRB) approved our interview protocol (Protocol E14-675) and all respondents gave informed verbal consent (written consent is problematic in former Soviet regions) as approved by the IRB.

We used a semi-structured questionnaire to interview ten herding families from three sites (**Fig 1**) about their fishing habits, fishing activity they observe, and status and conservation of fish in the lake (**S1 Appendix**). The first household at each site was selected opportunistically and additional households were recommended by this family based on proximity and availability. This “snowball sampling” method is commonly used to find respondents in isolated or hard-to-access groups [25]. There was no indication that recommendations were biased towards fishing or non-fishing households. We interviewed seven male and three female heads of household. Family and herd sizes ranged from 3–7 people and 4–630 animals, respectively.

We used a different semi-structured questionnaire to interview five park rangers, including the head ranger, from 5 of 17 ranger districts (**Fig 1**) about the frequency and character of illegal fishing, actions taken against illegal fishers, and status and conservation of fish in the lake (**S2 Appendix**). The interviewed rangers were male and had worked as rangers for 3–15 years. They were responsible for districts that varied in area (22–398 ha) and number of families (32–1,264 families).

### Biological sampling, gillnet catch efficiency, and population trends

We used fish monitoring data to estimate catch rates for gillnet mesh sizes used by fishers and to evaluate changes in fish population abundance and body size.

The Rutgers University Animal Care and Facilities Committee approved our fish sampling protocol (Protocol 11–005). Permission to conduct field research (Permit 6/445) was granted by the Mongolian Ministry of Environment and Green Development (MEGD). In July 2009 and 2011–13, we set two monofilament horizontal gillnets at seven of the ten surveyed sites (**Fig 1**). Both gillnets were 2 m deep and 20 m long with 4 m panels of 2.54, 3.81, 5.08, 6.35, and 7.62 cm bar mesh. They were set at least 100 m apart, perpendicular to shore, using a stationary bottom set in water < 10 m deep, and were fished overnight (8.5–10.5 hr) at each location. Captured fish were identified and measured to the nearest millimeter in total length. Weights for fish without weight measurements were estimated using length-weight parameters derived from our data (**S2 Fig**).

Vulnerability of fish to gillnets can vary depending on species, body size, and mesh size. We calculated catch-per-unit-of-effort (CPUE) for each gillnet panel in terms of count and biomass (#/kg 10 m^-1^ night^-1^) to determine species-specific and overall catch rates for each mesh size. We also calculated the species-specific CPUE of each gillnet set in terms of count and biomass (#/kg night^-1^) and used linear mixed effects models to examine changes in species-specific abundance from 2009–13 while accounting for sampling site as a random effect on the model intercepts. Decreases in body size can be a useful indicator of fishing impacts when changes in abundance cannot be accurately assessed [26]. Therefore, we also used linear mixed effects models to examine changes in species-specific body size (length/weight) from 2009–13. P-values were generated through likelihood ratio tests of the full models and null ‘intercept only’ models. All analyses were performed in R version 3.2.0 [27] and mixed effects models were fit using the *lme4* package [28].

### Potential population level impacts on Hovsgol grayling

We used methods commonly used in data-poor fisheries management to estimate the maximum sustainable yield (MSY) for Hovsgol grayling and evaluate the likelihood that illegal gillnet fishing could approach or exceed this threshold. Fishing at a rate greater than that which results in MSY is a common definition of overfishing [29].

Meta-analyses have shown that fish life history traits can be used to estimate natural mortality rates [30], which can in turn be used to estimate F_MSY_ [31], the fishing mortality rate resulting in MSY. We estimated the Hovsgol grayling natural mortality rate (*M*) using three separate life history invariant approaches (**Table 1**) and applied the Zhou et al. [31] method to estimate F_MSY_ as *0*.87*M. We used a length-converted catch curve analysis [32] to calculate total mortality (total mortality = fishing mortality + natural mortality) to place an upper limit on possible natural mortality rates and estimate current fishing mortality rates. More details on the mortality estimation methods are provided in **S3 Appendix**.

**Table 1:** Natural mortality rates estimated by life history invariant methods and estimates of the effort required to exceed the sustainable harvest associated with each mortality rate.

						# nights	# fishers	% families
Method	Formula^1^	M	F^2^	F_MSY_ ^3^	MSY (kg)^4^	required^5^	required^6^	participating^7^
Hoenig_nls_ from Then et al. [56]	4.899 * t_max_^-0.916^	0.37	0.06	0.32	330,869	22,058	220.6	14.6%
Pauly_nls-T_ from Then et al. [56]	4.118 * K^0.73^ * Linf^-0.33^	0.27	0.15	0.24	255,285	17,019	170.2	11.3%
Gunderson [57]	1.79 * GSI	0.30	0.12	0.26	279,557	18,637	186.4	12.3%

^1^ See **S6 Fig** for life history traits used in analysis.

^2^ *F = Z–M*, where Z is 0.42 from the length-converted catch curve analysis (**S7 Fig**).

^3^ *F*_*MSY*_ *= 0*.87 * M, from Zhou et al. [31].

^4^ *MSY = (1- exp(-F*_*MSY*_ )) * BIOMASS, where Hovsgol grayling biomass is 1,214,400 kg based on Ahrenstorff et al. [21].

^5^ Number of nights required to reach MSY assuming fishers use 50-m of optimal mesh gillnet each night (15 kg grayling night^-1^).

^6^ Number of fishers required to reach MSY assuming each fisher uses 50-m of optimal mesh gillnet 100 nights per year.

^7^ Percentage of families participating in the fishery assuming a resident population of 5,440 and average family size of 3.6 people per household (1,511 families; NSOM [19]).

We then calculated MSY for each F_MSY_ estimate using the Ahrenstorff et al. [21] hydroacoustic biomass estimate for Hovsgol grayling (4.4 ± 0.9 kg ha^-1^) and estimated the number of nights of gillnet fishing required to reach each MSY assuming fishers use 50-m gillnets with 2.54-cm mesh, the optimal mesh size for targeting grayling (~15 kg grayling night^-1^; see *Gillnet catch efficiency*results). Finally, we estimated the number of fishers required to achieve each MSY assuming fishers use 50-m of gillnet 100 nights year^-1^. These assumptions seem reasonable given the number of nets used by observed and self-reported fishers and reports that fishing continues throughout the winter (see *Interviews with herders and rangers*results).

## Results

### Surveys for derelict fishing gear

**Fig 2:**
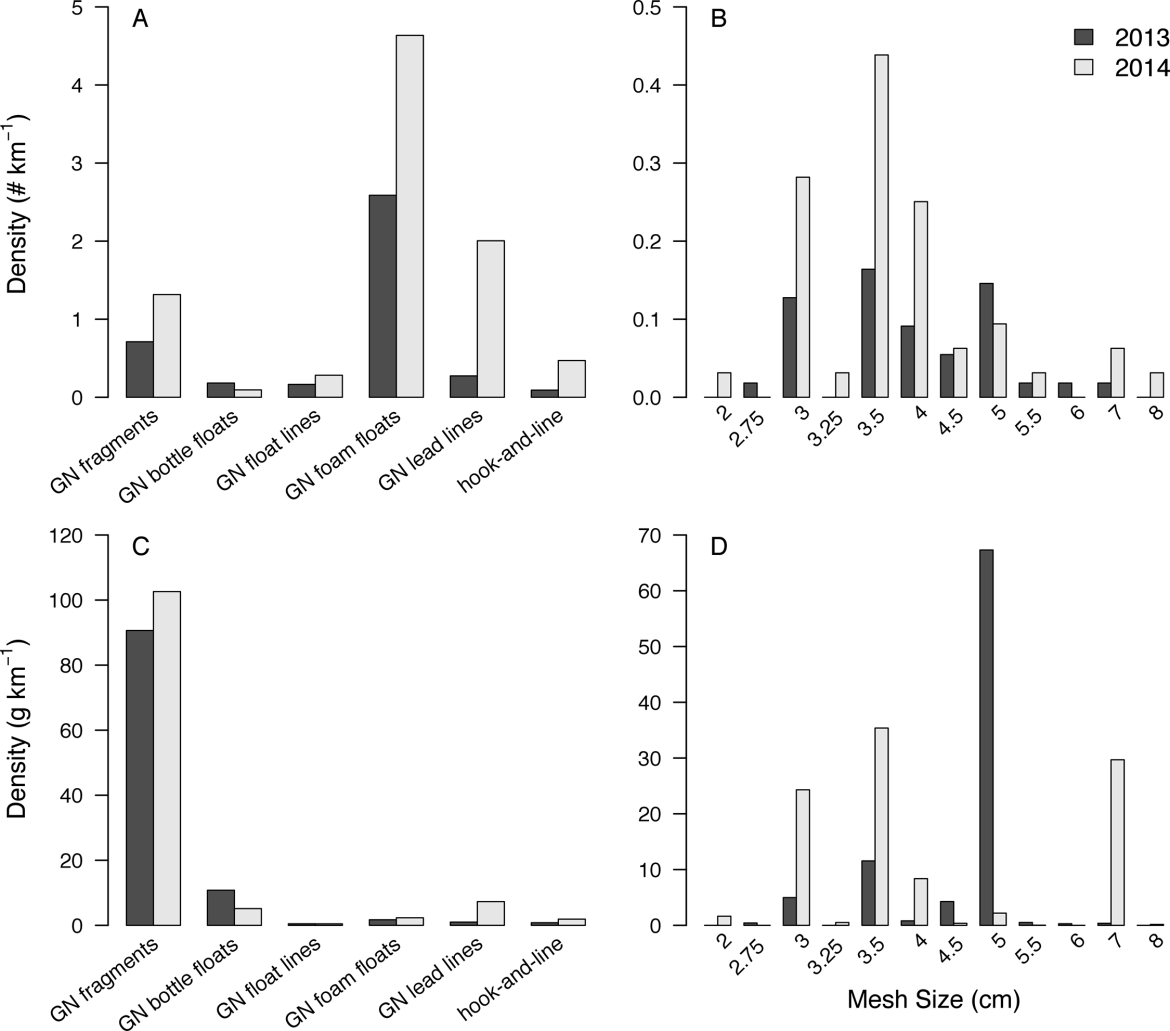
Average density of derelict fishing gear by category (GN = gillnet material) and derelict gillnet fragments by mesh size in count and weight. Bars indicate average densities among the 2013 (dark grey, n_sites_ = 10, n_transects_ = 14) and 2014 shoreline transects (light grey, n_sites_/n_transects_ = 7) weighted by transect length. Panels **A** and **B** indicate density in count (# km^-1^) and Panels **C** and **D** indicate density in weight (g km^-1^). Note variable y-axis scales.

A total of 220 (5.78 kg) and 281 (3.82 kg) pieces of derelict fishing gear were collected in the 2013 and 2014 surveys, respectively. Fishing gear comprised 25% of the total weight of plastic debris observed during the 2013 surveys [24]. Derelict gillnet material, the majority of fishing gear found in both years (**Fig 2**), was found in all but two 2013 transects and all 2014 transects (**Fig 1**). Foam floats were the most abundant gillnet debris items by count, likely due to their ability to separate from nets and disperse widely; gillnet fragments were the most abundant gillnet debris items by weight, likely due to their large size and heavy lead lines. Gillnet fragments ranged from 2–8 cm in mesh size with 3–4 cm mesh being the most common by both count and weight (**Fig 2**). All six active gillnets observed had 3.0 cm mesh. The density of derelict gillnet material varied among transects, but in both years, Site 7, the most remote and difficult to access site, had the lowest density of gillnet material and Site 10 (Har Us), the primary location of the spring spawning migration fishery, had the highest density of gillnet material. The density of derelict gillnet material in resurveyed sites was higher in 2014 than 2013 at all but Site 7 suggesting that illegal fishing may be increasing (**Fig 1**).

### Interviews with herders

All of the interviewed herding families (n = 10) reported fishing and observing others fishing (**S4 Table**). Families on the eastern shore reported fishing with gillnets repeatedly throughout the year and during the spring grayling spawning migration. They also reported observing commercial gillnet fishers from Hatgal during the winter and during the spring spawning migration, and they reported finding enforcement ineffective. In contrast, families on the northwestern shore reported fishing with rods or by hand only once per spring spawning migration. They reported no commercial fishing activity and found enforcement effective. All of the families reported that Russian visitors fish recreationally year-round but especially in winter with ice fishing rods and gillnets (**S4 Table**).

All of the families reported fishing primarily for Hovsgol grayling and primarily for household consumption; only one family from the eastern shore reported selling fish (**S4 Table**). Families reported fishing primarily during the spawning migration because (1) grayling soup is healthy after the long winter; (2) fish are more abundant and easier to catch than any other time; (3) herders are too busy to fish, or they live away from the lake, the rest of the year; (4) cooking grayling soup interferes with milk production, their principal food source; and (5) eating grayling allows them to delay the slaughtering of herd animals until they have had time to fatten.

Nearly all of the interviewed herders stated that fish population sizes have decreased dramatically (**S4 Table**). Many recalled that migrating fish were once so numerous that the rivers appeared to “be only fish and no water.” Most of the herders also asserted that fish body sizes have decreased and that large lenok and burbot have become especially rare (**S4 Table**). The herders stated that “local people should protect the lake and fish” but offered few concrete ideas for achieving this objective (**S4 Table**).

### Interviews with rangers

The rangers reported that recreational, commercial, and subsistence fishing all occur in LHNP (**S5 Table**). The rangers agreed that the majority of recreational fishers are non-local Mongolians or foreigners who fish with rods primarily in summer but also through the ice in winter. The rangers reported that recreational fishers are generally permitted and compliant with the law. All but one ranger reported that local Mongolians use gillnets to target Hovsgol grayling and lenok for subsistence or commercial purposes (**S5 Table**). The rangers reported that subsistence fishers fish almost exclusively at river mouths during the spring spawning migration and that commercial fishers come predominantly from Hatgal due to that town’s proximity to the developed southwestern shore and the city of Mörön. The rangers asserted that the town of Hankh is too remote and undeveloped for commercial fishing to be viable. The rangers reported that commercial gillnet fishing occurs year-round and that fishing when the lake is freezing, thawing, or entirely frozen may even be preferred (**S5 Table**).

The rangers were divided on the status of fish in the lake: three rangers reported that fish population sizes are decreasing and two rangers reported that they are increasing (**S5 Table**). The rangers who reported fish population sizes to be decreasing reported that lenok have become especially rare. The majority of rangers reported that fish body sizes have not changed (**S5 Table**). The rangers were also divided on the best approach to conservation. The head ranger asserted that the native Great Cormorant (*Phalacrocorax carbo*) population is the primary threat to fish and that their population must be controlled. Another ranger suggested that grayling die naturally after the spring spawning migration (an assertion that is not supported by the scientific literature) and that these migrations must therefore be prevented. The remaining rangers emphasized the importance of improved enforcement during the spawning migration (**S5 Table**).

The rangers offered a detailed description of fishing at Har Us mineral spring (Site 10), the primary location of the spring grayling spawning migration fishery. Mineral springs are culturally important to Mongolians and visiting this spring in May-June is a longstanding social tradition. Rangers are instructed not to enforce the gillnet ban on fishers at Har Us during this time. The rangers reported that over 570 people visited the spring in 2013 and set a total of 60–100 nets per day with an average catch of 50–70 grayling per net. They estimated that 3,600 grayling were caught per day during peak migration (Jun 7–12) and 1,000–1,500 grayling per day from May 30-Jun 6 and Jun 13–24. Based on this report, we estimate that the Har Us fishery removes ~33,000 fish annually.

### Gillnet catch efficiency and population trends

**Fig 3:**
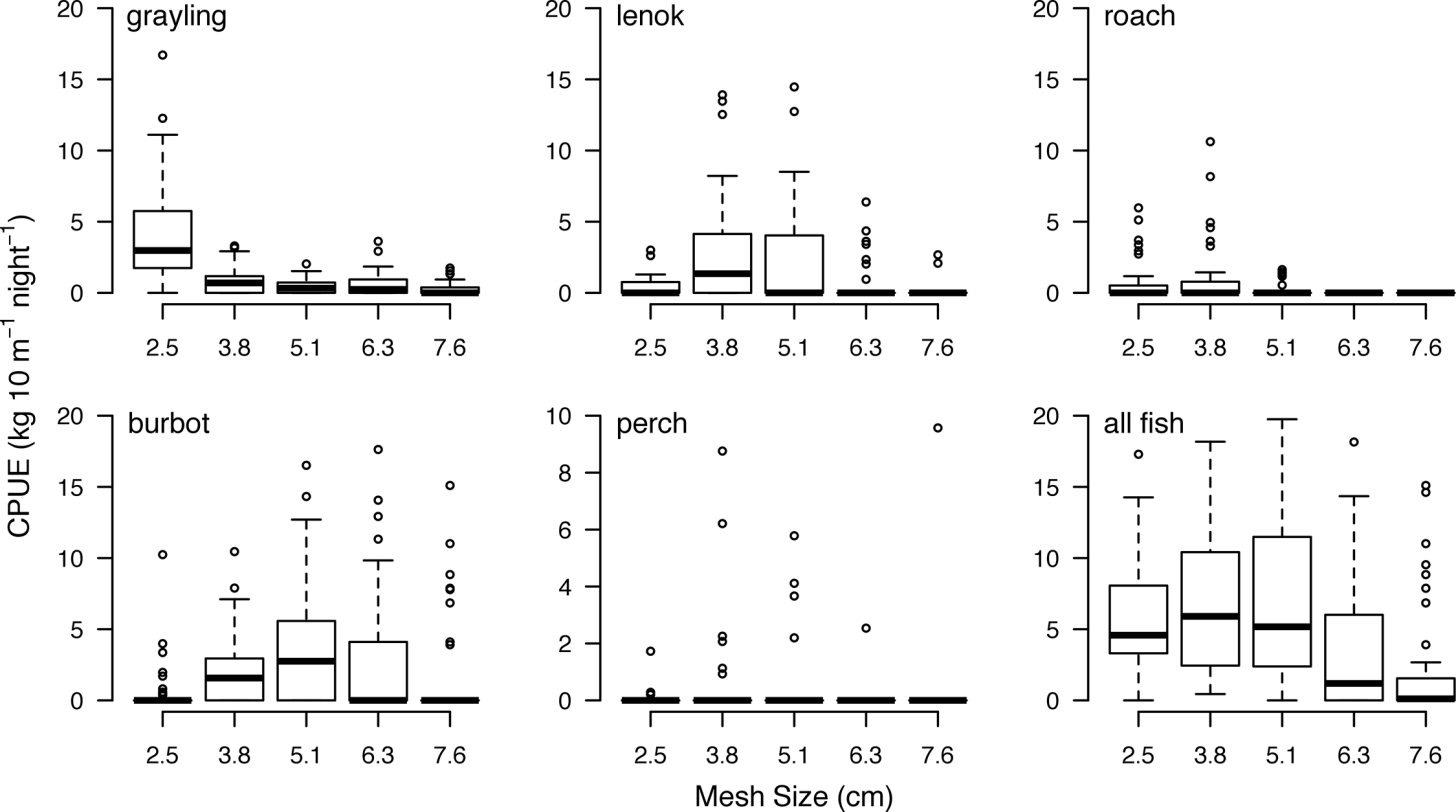
Catch-per-unit-of-effort (CPUE; kg 10 m^-1^ night^-1^) by mesh size for the five most abundant species in gillnet catches and the sum of their weight. Data from the two 5-panel sequential mesh gillnets used at seven sites in 2009 and 2011–2013 (14 sets yr^-1^, 56 sets total). Boxplots indicate median (heavy black line), interquartile range (IQR; box), 1.5 times the IQR (whiskers), and extreme values (open circles). Note variable y-axis scales.

The 2.54-cm mesh in our survey gillnets maximized total nightly catch by numbers because it maximized the catch of the abundant Hovsgol grayling (**S3 Fig**). The 3.81- and 5.08-cm mesh sizes showed similar catch rates and maximized total nightly catch by biomass because they maximized the catch of larger-bodied lenok and burbot (**Fig 3**); however, the median nightly catch biomass of the 2.54-cm mesh was comparable to those of the 3.08- and 5.81-cm mesh and the 2.54-cm mesh captured fish during every gillnet set, while the larger mesh sizes were often observed empty.

**Fig 4:**
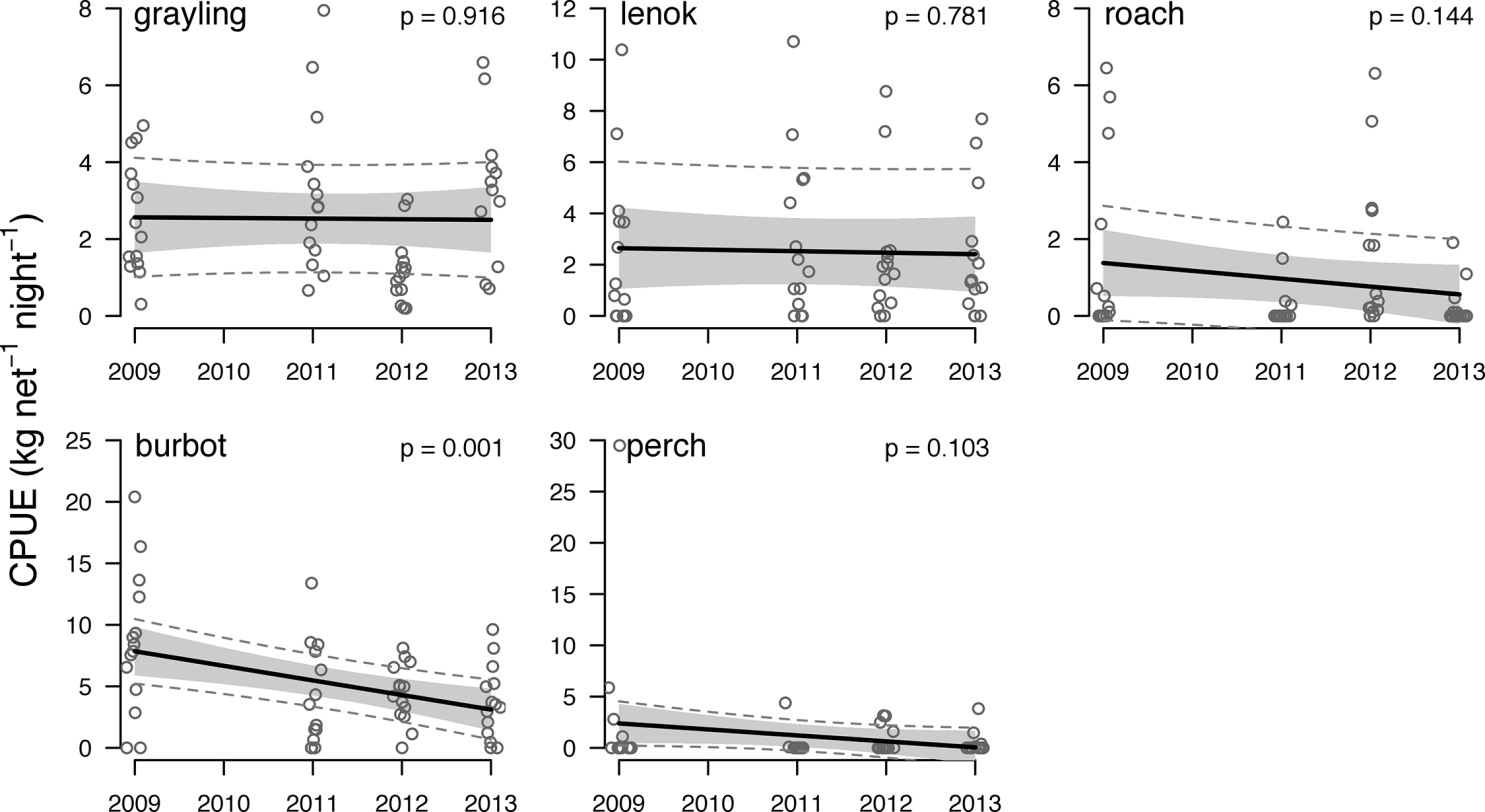
Trends in the abundance of the five most abundant fish species in gillnet catches from 2009–2013. Points indicate the CPUE (kg net^-1^ night^-1^) of each 5-panel sequential mesh gillnet set (2 nets site^-1^ x 7 sites yr^-1^ = 14 sets yr^-1^). Dark lines indicate linear mixed effects regressions fit to the catch data, gray shading indicates the confidence interval for each regression, and dashed lines indicate the prediction interval for the data. P-values are indicated in the upper right corner of each panel. Points are jittered around year for display. Note variable y-axis scales.

**Fig 5:**
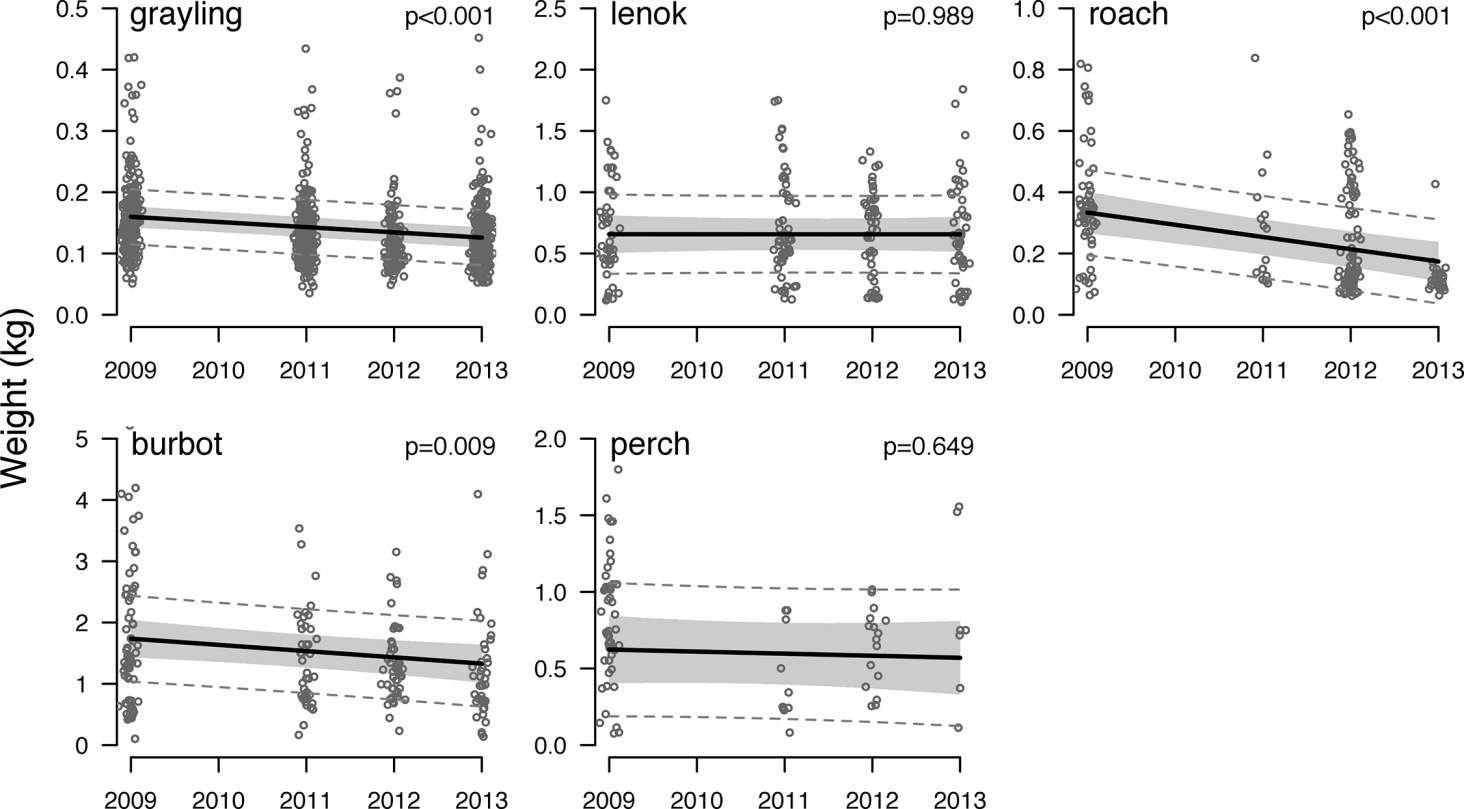
Trends in the body size of the five most abundant fish species in gillnet catches from 2009–2013. Points indicate the weight (kg) of every fish caught in gillnet sets that year (2 nets site^-1^ x 7 sites yr^-1^ = 14 sets yr^-1^). Dark lines indicate linear mixed effects regressions fit to the catch data, gray shading indicates the confidence interval for each regression, and dashed lines indicate the prediction interval for the data. P-values are indicated in the upper right corner of each panel. Points are jittered around year for display. Note variable y-axis scales.

Analysis of the biological monitoring data identified significant reductions in body size for three species over the sampling period (2009–13), but a significant change in CPUE for only one species. Linear mixed effects regression on species-specific CPUE indicates that only burbot population abundance decreased significantly from 2009–13 (**Fig 4; S4 Fig**). Linear mixed effects regression on body size indicates that grayling, roach, and burbot body size decreased significantly from 2009–13 (**Fig 5; S5 Fig**). The abundance and body size of other species remained constant.

### Potential population level impacts on Hovsgol grayling

Estimates of Hovsgol grayling natural mortality (*M*) ranged 0.25–0.37 (**Table 1**). A total mortality estimate of 0.42 (**S7 Fig**) implies fishing moralities of 0.06–0.15, all of which are less than their associated F_MSY_ estimates (**Table 1**). The F_MSY_ estimates imply MSY values of ~255–331 metric tons yr^-1^, which could be achieved in ~17,000–22,000 nights of fishing with 50-m optimal mesh gillnets (**Table 1**). Although these estimates seem large for a low-density resident population, they could be achieved by 170–220 fishers using 50-m of optimal mesh gillnet 100 nights year^-1^ (roughly twice per week). With an estimated permanent population of 5,440 in LHNP and average family size of 3.6 people [19], this effort could be attained if 11.3–14.6% of families participated in the fishery (**Table 1**). Alternatively, this effort could be attained if every family living in the park fished with 50-m of optimal mesh gillnet 11.3–14.6 nights per year.

## Discussion

Knowledge of illegal fishing in Lake Hovsgol National Park (LHNP) has been anecdotal and limited in its usefulness to managers, but with a mixed-method approach, we have empirically described the extent, character, and motivations of illegal fishing and its potential impact on the lake’s fish populations.

Our mixed-method approach reveals a fuller understanding of illegal fishing in LHNP than using a single method alone. Each method validates, contextualizes, and builds upon the others to construct a consistent story for a complex fishery: **(1) surveys for derelict fishing gear** quantitatively describe the extent, location, and methods of fishing: gillnet fishing is widespread and increasing and fishers generally use 3–4 cm mesh gillnet; **(2) interviews with herders and park rangers** contextualize these results by qualitatively describing the motivations of fishers, character of fishing, and status of fish in the lake: many residents gillnet fish for subsistence during the spring grayling spawning migration, some residents gillnet fish commercially year-round, and fish population sizes are decreasing; **(3) biological monitoring** documents the vulnerability of fish to gillnets as well as population-level trends in fish abundance and body size: the gillnet mesh sizes used by fishers efficiently target Hovsgol grayling and grayling, burbot, and roach exhibit negative population-level trends; and **(4) data-poor stock assessment analyses** demonstrate that plausible levels of fishing effort by Lake Hovsgol residents using gillnets have the capacity to result in overexploitation of the Hovsgol grayling population. Though seemingly intuitive, the use of multiple methods to quantify and characterize illegal resource use has been rare and should be more widely used by conservation scientists and resource managers [4,5].

Our surveys for derelict fishing gear are an improvement to previous studies because we use repeated surveys to measure re-accumulation rates and biological monitoring data to evaluate the vulnerability of fish to the gear observed in surveys. The majority of studies have focused on comparing the density of derelict gear inside and outside marine reserves for quantifying non-compliance and fail to measure or report accumulation rates (e.g., [9–11]). A few studies have measured the accumulation rates of derelict gear among habitat types to inform cleanup efforts but have not used the results to understand non-compliance (e.g., [8,33,34]). Only Williamson et al. [14] and the present study have linked these objectives and used both the density and re-accumulation rate of derelict fishing gear to evaluate temporal and spatial trends in non-compliance. By measuring re-accumulation, we show not only that the observed gillnet was used recently and does not pre-date the ban on gillnet fishing, but also that gillnet fishing is becoming increasingly common. Neither Williamson et al. [14] or our study properly control for the influence of habitat characteristics (e.g., shore/bottom cover or wind/wave exposure) on accumulation and future studies must consider these covariates when identifying hotspots of illegal fishing.

Although our interview method likely underestimates the rate of non-compliance [35,36], it provides a relative description of the frequency of illegal fishing and important information about the motivations for non-compliance, which cannot be gained using other respondent-based approaches [4]. The biases and limitations of direct questioning (DQ) can be reduced when researchers have long-standing relationships with the community [37,38] or by interviewing multiple stakeholders [37,39]. In our study, this likely contributes to the discrepancy in personal fishing habits reported by herders on the eastern and western shores. Whereas eastern shore herders, with whom we have long partnerships, reported frequent gillnet use, western shore herders reported fishing by hook and line or by hand only. Although this may reflect real geographic differences, it may also reflect social desirability bias [40], as western shore herders might be less comfortable revealing sensitive information to us. In our study, this bias is partially corrected by interviewing multiple stakeholders and by inquiring about observed illegal behavior [37,39]. For example, herders were more likely than park rangers to characterize enforcement as ineffective and park rangers were more likely than herders to describe illegal fishing. Similarly, although some respondents were likely to underreport personal fishing, they may not be as likely to underreport observed fishing by others.

Because of these biases, recent papers promote the randomized response (RRT; [41]) and item count techniques (ICT; [42]) over DQ for quantifying non-compliance [36,43–45], but we argue that DQ more easily and fully reveals the motivations for non-compliance [4], which is essential information for successful management [46]. RRT and ICT incentivize honest responses about illegal behavior by protecting anonymity and generally generate more accurate estimates of the proportion of the sample population engaging in illegal behavior [35,36]; however, these approaches require large sample sizes and prevent researchers from implicitly discerning motivations for non-compliance by linking behaviors with covariates or from explicitly inquiring about the motivations for non-compliance [45]. DQ, on the other hand, allows researchers to inquire about the motivations for non-compliance, importance of natural resources to culture or livelihood, and desire for changes to management rules. Managers must consider the socioeconomic functions of resource use and DQ should remain in the conservation science toolbox.

Although the population-level impacts observed in our biological monitoring data cannot necessarily be attributed to illegal fishing, they indicate the importance of improving fisheries management in LHNP, especially given the feasibility for gillnet fishers to overexploit the Hovsgol grayling population, as indicated by the data-poor stock assessment analysis. These calculations represent a simplification of population dynamics made necessary by the lack of time series of fishery removals or estimates of biological parameters needed for more complex data-poor assessment methods [47]. However, our indirect estimates of *M* for Hovsgol grayling are similar to direct estimates of *M* for Arctic grayling (*T*. *arcticus*), a close relative (0.29 average; **S3 Table**). Furthermore, all of our MSY estimates indicate that overexploitation is possible even with only a small percentage of the population participating in the fishery using gillnets, an inexpensive and widely available fishing gear. The threat of overexploitation is not unrealistic given that grayling, as a taxonomic group, can be susceptible to anthropogenic influences as has been seen with the extirpation of many North American Arctic grayling populations in Montana and Wyoming [48]. Salmonids are vulnerable to exploitation and other disruptions during their spring spawning migrations [49] and managers must carefully consider the value and impact of the spring spawning migration fishery.

The results of our mixed-method approach indicate that illegal fishing is a problem in Lake Hovsgol but that fish also serve an important socioeconomic function. An effective management system will need to incorporate the needs of local people as well as address the synergistic pressures of climate change, water pollution, increasing tourism, and invasive species on LHNP’s fish populations. In the last 40 years, regional air temperatures have increased 2.1°C [50], a rate of warming more than three times faster than the global average [51], which has prompted the drying of many of Lake Hovsgol’s previously reliable streams and loss of grayling spawning habitat [16,17]. Increasing tourism may result in increased fishing pressure, habitat destruction, water pollution, and invasive species introductions without proper management. Lake Hovsgol is already heavily polluted with household trash and will only become more polluted with additional strains on its inadequate waste management system [24]. Although no invasive species have established to date, the successful introduction of a new fish or aquatic plant species could alter this otherwise intact ecosystem [52].

Fishing, historically uncommon in Mongolia’s pastoralist culture, may be gaining prevalence as a new source of food, income, or recreation, especially as climate change makes herding more difficult [53] and urban Mongolians acquire more globalized tastes in food and leisure [54]. At the same time, Mongolia aims to protect 30% of the country by 2030, more than doubling the area currently under protection [55]. These trends forecast continued conflicts between economic and conservation objectives and the way in which these conflicts are resolved or ignored in the iconic LHNP could shape future protected area management in the country.

## Supporting Information

S1 Table Large-bodied fish species in Lake Hovsgol, Mongolia and their historic catch*, market price^†^, and fine per illegally caught fish^‡^. (DOCX)

S2 Table Life history invariant methods selected for estimating Hovsgol grayling natural mortality rate. (DOCX)

S3 Table Arctic grayling (*Thymallus arcticus*) natural mortality rates reported in the literature. (DOCX)

S4 Table Responses of ten herding families interviewed about their personal fishing habits, fishing activity they observe, and status and conservation of fish in the lake. (DOCX)

S5 Table Responses of five park rangers interviewed about the frequency and character of illegal fishing, actions taken against illegal fishers, and status and conservation of fish in the lake. (DOCX)

S1 Fig Diagram of a typical Mongolian horizontal gillnet and its components. (PNG)

S2 Fig Length-weight relationships for the five most abundant fish species in gillnet catches in Lake Hovsgol. Note variable y-axis scales.
(PNG)

S3 Fig Catch-per-unit-of-effort (CPUE; # 10 m^-1^ night^-1^) by mesh size for the five most abundant species in gillnet catches and the sum of their catch. Data from the two 5-panel sequential mesh gillnets used at seven sites in 2009 and 2011–2013 (14 sets yr^-1^ x 4 yr = 56 sets total). Boxplots indicate median (heavy black line), interquartile range (IQR; box), 1.5 times the IQR (whiskers), and extreme values (open circles). Note variable y-axis scales.
(PNG)

S4 Fig Trends in the abundance of the five most abundant fish species in gillnet catches from 2009–2013. Points indicate the CPUE (# net^-1^ night^-1^) of each 5-panel sequential mesh gillnet set (2 nets site^-1^ x 7 sites yr^-1^ = 14 sets yr^-1^). Dark lines indicate linear mixed effects regressions fit to the catch data, gray shading indicates the confidence interval for each regression, and dashed lines indicate the prediction interval for the data. Points are jittered around year for display. P-values are indicated in the upper right corner of each panel. Note variable y-axis scales.
(PNG)

S5 Fig Trends in the body size of the five most abundant fish species in gillnet catches from 2009–2013. Points indicate the total length (mm) of every fish caught in gillnet sets that year (2 nets site^-1^ x 7 sites yr^-1^ = 14 sets yr^-1^). Dark lines indicate linear mixed effects regressions fit to the catch data, gray shading indicates the confidence interval for each regression, and dashed lines indicate the prediction interval for the data. P-values are indicated in the upper right corner of each panel. Points are jittered around year for display. Note variable y-axis scales.
(PNG)

S6 Fig Estimates of the life history characteristics used to calculate natural mortality (*M*) for Hovsgol grayling. See **Table 1**for *M* estimation methods and results. In **(A),** *L*_*inf*_, *K*, and *t*_*max*_ were estimated from aged otoliths and a von Bertalanffy growth model (black line) fit through the observed age-size relationship and origin (Tsogotsaikhan et al. in review). In **(B),** *GSI* was estimated as the mean gonadosomatic index (GSI) for all observed grayling (Jensen, unpublished data). In **(B),** the black line indicates a linear regression fit and the grey shading indicates the confidence interval for the regression. Life history characteristics are marked and labeled in both panels.
(PNG)

S7 Fig The (A) length and (B) length-converted age structure of the Hovsgol grayling population. The length strucutre was observed in the Ahrenstorff et al. (2012) hydroacoustic surveys. In **(B)**, the solid black line indicates a linear regression fit to the log-transformed trailing arm of the age structure. The dashed black lines indicate the confidence interval for the regression. Z is equal to the negative slope of the regression.
(PNG)

S1 Appendix Herder interview questionnaire. (DOCX)

S2 Appendix Park ranger interview questionnaire. (DOCX)

S3 Appendix Total and natural mortality estimation. (DOCX)

S4 Appendix Supplemental references. (DOCX)
