# Correction: A Mixed-Method Approach for Quantifying Illegal Fishing and Its Impact on an Endangered Fish Species

**DOI:** 10.1371/journal.pone.0148007

**Published:** 2016-01-25

**Authors:** Christopher M. Free, Olaf P. Jensen, Bud Mendsaikhan

The following information is missing from the Funding section: This publication (NJSG-16-894) is the result of research sponsored by the New Jersey Sea Grant Consortium (NJSGC) with funds from the National Oceanic and Atmospheric Administration (NOAA) Office of Sea Grant, U.S. Department of Commerce, under NOAA grant number NA13OAR4170114 and the NJSGC. The statements, findings, conclusions, and recommendations are those of the author(s) and do not necessarily reflect the views of the NJSGC or the U.S. Department of Commerce.

## References

[1] Free CM, Jensen OP, Mendsaikhan B (2015) A Mixed-Method Approach for Quantifying Illegal Fishing and Its Impact on an Endangered Fish Species. PLoS ONE 10(12): e0143960 doi: 10.1371/journal.pone.0143960 26625154 10.1371/journal.pone.0143960 PMC4666464

