# Supplementary material for: A Mixed-Method Approach for Quantifying Illegal Fishing and Its Impact on an Endangered Fish Species
Source: PLoS One. 2015 Dec 1;10(12):e0143960. doi: 10.1371/journal.pone.0143960 (PMC4666464; doi:10.1371/journal.pone.0143960)
Supplement: S1 Table — (DOCX) [file pone.0143960.s001.docx]

**S1 Table.** Large-bodied fish species in Lake Hovsgol, Mongolia and their historic catch*, market price^†^, and fine per illegally caught fish^‡^.

|  |  |  | **Historic** | **Market** | **Fine** |
| --- | --- | --- | --- | --- | --- |
| **Family** | **Scientific Name** | **Common Name** | **Catch (t yr^-1^)** | **Price (₮)** | **Amount (₮)** |
| Thymallidae | *Thymallus nigrescens* | Hovsgol grayling | 100 | 1,500 | 5,000 |
| Lotiidae | *Lota lota* | Burbot | 50 | 15,000 | 13,000 |
| Percidae | *Perca fluviatilis* | Eurasian perch | 50 | --- | 5000 |
| Salmonidae | *Brachymystax lenok* | Lenok | 24 | --- | 17,000 |
| Cyprinidae | *Rutilus rutilus* | Roach | --- | --- | 5000 |

* From Dulmaa 1999.

^†^ Mendsaikhan personal observation. ₮ denotes Mongolian tugrik. In July 2014, US$1 was approximately ₮1825.

^‡^ Established by Protocol #23 (2011) of the Mongolian Law on Hunting (2000) according to ecological evaluation.
