# Supplementary material for: A Mixed-Method Approach for Quantifying Illegal Fishing and Its Impact on an Endangered Fish Species
Source: PLoS One. 2015 Dec 1;10(12):e0143960. doi: 10.1371/journal.pone.0143960 (PMC4666464; doi:10.1371/journal.pone.0143960)
Supplement: S2 Table — (DOCX) [file pone.0143960.s002.docx]

**S2 Table.** Life history invariant methods selected for estimating Hovsgol grayling natural mortality rate.

| **Method** | | **Formula***^†^ | **Used?** | **Comments** |
| --- | --- | --- | --- | --- |
| 1 | Hoenig 1983 | M ~ t_max_ | YES | Used updated regression from Then et al. 2014 (based on 226 populations) |
| 2 | Pauly 1980 | M ~ L_inf_/W_inf_, K, (T) | YES | Used updated regression from Then et al. 2014 (based on 218 populations) |
| 3 | Gunderson 1997 | M ~ GSI | YES | Used regression from Gunderson 1997 (based on 28 populations) |
| 4 | Tanaka 1960 (rule of thumb) | M ~ t_max_ | NO | Does not perform better than Hoenig's estimator (Then et al. 2014) |
| 5 | Sekharan 1975 | M ~ t_max_ | NO | Does not perform better than Hoenig's estimator (Then et al. 2014) |
| 6 | Griffiths & Harrod 2007 | M ~ L_inf_/W_inf_, K | NO | Does not perform better than Pauly's estimator (Then et al. 2014) |
| 7 | Jensen 1996 - K | M ~ K | NO | Does not perform better than Pauly's estimator (Then et al. 2014) |
| 8 | Jensen 2001 - K, T | M ~ K, T | NO | Does not perform better than Pauly's estimator (Then et al. 2014) |
| 9 | Roff 1984 - L_inf_, K, L_mat_ | M ~ L_inf_, K, L_mat_ | NO | Requires L_mat_ |
| 10 | Charnov & Berrigan 1990 | M ~ t_mat_ | NO | Requires validated t_mat_ |
| 11 | Rikhter & Efanov 1976 - t_mat_ | M ~ t_mat_ | NO | Requires validated t_mat_ |
| 12 | Jensen 1996 - t_mat_ | M ~ t_mat_ | NO | Requires validated t_mat_ |
| 13 | Roff 1984 - K, t_mat_ | M ~ K, t_mat_ | NO | Requires validated t_mat_ |
| 14 | Rikhter & Efanov 1976 - β, K, t_0_, t_mat_ | M ~ β, K, t_0_, t_mat_ | NO | Requires validated t_mat_ |
| 15 | Alverson & Carney 1975 | M ~ K, t_max_ | NO | Requires data from unexploited era |
| 16 | Zhang & Megrey 2006 | M ~ β, K, t_0_, t_mb_ | NO | Requires data from unexploited era; requires validated t_mb_; requires t_0_ fit |
| 17 | Cubillos et al. 1999 | M ~ K, t_0_ | NO | Requires t_0_ fit [Tsogotsaikhan et al. (in review) fix t_0_ at 0] |
| 18 | Frisk et al. 2001 | M ~ K/t_max_ | NO | For elasmobranchs |
| 19 | Ralston 1987 | M ~ K | NO | For Lutjanid snappers and Serranid groupers |
| 20 | Djabali et al. 1994 | M ~ L_inf_/W_inf_, K | NO | For Mediterranean Sea fish; uses estimated M values; performs poorly |
| 21 | Alagaraja 1984 | M ~ L_inf_, K, t_0_ | NO | Dubious assumptions about age at L_inf_; requires t_0_ fit |
| 22 | Groeneveld 2000 | M ~ L_inf_, K, L_mat_ | NO | Estimates cannot be replicated; severe overestimation; requires L_mat_ |
| 23 | Lorenzen 1996 | M_w_ ~ w | NO | M varies with individual weight |
| 24 | Peterson & Wroblewski 1984 | M_w_ ~ w | NO | M varies with individual weight; includes non-fish animals |
| 25 | Ursin 1967 | M_w_ ~ w | NO | M varies with individual weight; severe underestimation |
| 26 | Jennings & Dulvy 2008 | M_w_ ~ T, w | NO | M varies with individual weight |
| 27 | Gislason et al. 2010 | M_L_ ~ L_inf_, K, L | NO | M varies with individual length |
| 28 | Chen & Watanabe 1989 | M_t_ ~ K, t_0_, t_s_, t | NO | M varies with individual age; requires extreme assumptions; performs poorly |
| 29 | Bayliff 1967 | Z ~ t_max_ | NO | Estimates Z; for Engraulidae (anchovies) with only 6 data points |
| 30 | Kenchington 2014 | Z ~ t_max_, t_c_, n_e_ | NO | Estimates Z; problems with proof (Then et al. 2014) |
| 31 | Beverton & Holt 1959 | Z ~ L_inf_, L_mean_, L_crit_ | NO | Estimates Z |

***** M = natural mortality rate; M_L_ = natural mortality rate at length L; M_t_ = natural mortality rate at age t; M_w_ = natural mortality rate at weight w; Z = total mortality rate

^†^ β = exponent of the length/weight relationship; GSI = gonadosomatic index (wet ovary weight over wet body weight); K = parameter of von Bertalanffy growth curve; L = fish length; L_crit_ = minimum length of individuals fully represented in the catch; L_inf_ = asymptotic fish length; L_mat_ = length at reproductive maturity; L_mean_ = mean length of individuals between L_crit_ and the maximum length in the catch; n_e_ = effective sample size; t = fish age; T = mean environmental temperature; t_0_ = parameter of the von Bertalanffy growth curve; t_c_ = youngest age fully represented in the catch; t_mat_ = age at reproductive maturity; t_max_ = maximum observed or assumed age; t_mb_ = age at which year-class achieves its maximum biomass in the absence of fishing; t_s_ = age at onset of senescence; w = fish weight; W_inf_ = asymptotic fish weight
