# Supplementary material for: A Mixed-Method Approach for Quantifying Illegal Fishing and Its Impact on an Endangered Fish Species
Source: PLoS One. 2015 Dec 1;10(12):e0143960. doi: 10.1371/journal.pone.0143960 (PMC4666464; doi:10.1371/journal.pone.0143960)
Supplement: S3 Table — (DOCX) [file pone.0143960.s003.docx]

**S3 Table.** Arctic grayling (*Thymallus arcticus*) natural mortality rates reported in the literature.

| **Study** | **Location** | **Time period** | **M** |
| --- | --- | --- | --- |
| Clark 1993 | Fielding Lake, AK, USA | 1986-1990 (4 yr) | 0.24 |
| Fleming 1995 | Piledriver Slough, AK, USA | 1993-1994 (1 yr) | 0.27 |
| Buzby & Deegan 2000 | Kupurak River, AK, USA | 1983-1998 (15 yr) | 0.28 |
| Clark 1992 | Chena River, AK, USA | 1979-1988 (9 yr) | 0.31 |
| Clark 1995 | Chena River, AK, USA | 1991-1994 (3 yr) | 0.34 |
|  |  | **Average:** | **0.29** |
