# Supplementary material for: A Mixed-Method Approach for Quantifying Illegal Fishing and Its Impact on an Endangered Fish Species
Source: PLoS One. 2015 Dec 1;10(12):e0143960. doi: 10.1371/journal.pone.0143960 (PMC4666464; doi:10.1371/journal.pone.0143960)
Supplement: S4 Table — (DOCX) [file pone.0143960.s004.docx]

**S4 Table.** Responses of ten herding families interviewed about their personal fishing habits, fishing activity they observe, and status and conservation of fish in the lake.

| **Question** | **Responses (out of 10 respondents)** | **Response notes** |
| --- | --- | --- |
| Does your family fish? | yes, often (3) | Herding families on the northwestern shore rarely fish (~once during the spawning migrations). Herding families on the northeastern shore fish more often. |
|  | yes, sometimes (2) |  |
|  | yes, rarely (5) |  |
|  | no response (0) |  |
| Why does your family fish?* | food (10) | Herding families fish primarily for food. Only one family sells fish when the rare opportunity arises. |
|  | income (1) |  |
|  | recreation (1) |  |
|  | no response (0) |  |
| When does your family fish?* | during the spring spawning migration only (7) | Herding families fish primarily during the spring spawning migration when grayling are easy to catch. |
|  | all-year but especially during the spring spawning migration (3) |  |
|  | no response (0) |  |
| What gear does your family use to fish?* | rod (2) | Herding families on the northwestern shore fish primarily with rods or by hand. Herding families on the northeastern shore fish primarily with gillnets. |
|  | gillnet (4) |  |
|  | hand catch (1) |  |
|  | no response (3) |  |
| What fish do you catch and eat?* | grayling (10) | Grayling are often used to make soup. Grayling soup is thought to be healthy after a long cold winter. |
|  | lenok (2) |  |
|  | burbot (2) |  |
|  | perch (1) |  |
|  | no response (0) |  |
| Who else do you see fishing?* | local Mongolians (8) | Local Mongolians fish for subsistence with rods and gillnets. Mongolians from Hatgal fish commercially with gillnets. Foreigners fish recreationally primarily with rods but sometimes with gillnets. |
|  | Mongolians from Hatgal (6) |  |
|  | foreigners (mainly Russians) (8) |  |
|  | no response (0) |  |
| Why do they fish?* | food (9) |  |
|  | income (5) |  |
|  | recreation (7) |  |
|  | no response (0) |  |
| What gear do they use to fish?* | rod (6) |  |
|  | gillnet (9) |  |
|  | no response (0) |  |
| When do they fish?* | spring (10) | Local people fishing for subsistence fish primarily during the spring spawning migration. Tourists fish primarily during summer and winter. Commercial fishers fish year-round. |
|  | summer (2) |  |
|  | fall (0) |  |
|  | winter (5) |  |
|  | no response (0) |  |
| Have fish populations increased, decreased, or remained the same? | increased (0) | Grayling spring spawning migrations are shorter and less intense. Lenok and burbot have become especially rare. |
|  | decreased (9) |  |
|  | remained the same (0) |  |
|  | doesn't know (1) |  |
|  | no response (0) |  |
| Have fish body sizes increased, decreased, or remained the same? | increased (0) | Large lenok and burbot have become especially rare. |
|  | decreased (6) |  |
|  | remained the same (1) |  |
|  | doesn't know (2) |  |
|  | no response (1) |  |
| What should be done to protect fish?* | improve enforcement (4) | Few concrete ideas were provided. Herding families on northwestern shore find enforcement to be effective. |
|  | control cormorant population (1) |  |
|  | local people should protect the lake (7) |  |
|  | no response (0) |  |

* Multiple responses possible for these questions.
