# Supplementary material for: A Mixed-Method Approach for Quantifying Illegal Fishing and Its Impact on an Endangered Fish Species
Source: PLoS One. 2015 Dec 1;10(12):e0143960. doi: 10.1371/journal.pone.0143960 (PMC4666464; doi:10.1371/journal.pone.0143960)
Supplement: S5 Table — (DOCX) [file pone.0143960.s005.docx]

**S5 Table.** Responses of five park rangers interviewed about the frequency and character of illegal fishing, actions taken against illegal fishers, and status and conservation of fish in the lake.

| **Question** | **Responses (out of 5 respondents)** | **Response notes** |
| --- | --- | --- |
| Who fishes?* | local Mongolians (5) | Non-local Mongolians and foreigners fish recreationally. Local Mongolians fish for food and/or income. Commercial fishers come primarily from Hatgal. |
|  | non-local Mongolians (3) |  |
|  | foreigners (3) |  |
|  | no response (0) |  |
| Why do people fish?* | food (5) |  |
|  | income (3) |  |
|  | recreation (4) |  |
|  | no response (0) |  |
| What gear do people use?* | rods (4) | Recreational fishers use rods. Subsistence and commercial fishers use gillnets. |
|  | nets (4) |  |
|  | no response (0) |  |
| When do people fish?* | spring (3) | Recreational rod fishers fish all year-round but mostly in summer. Commercial and subsistence gillnet fishers fish mostly in the spring during the grayling migration and fall, winter, and spring when the lake is freezing or frozen. |
|  | summer (3) |  |
|  | fall (3) |  |
|  | winter (4) |  |
|  | no response (0) |  |
| What fish do people catch and eat?* | grayling (5) | Grayling and lenok are the primary targets of all types of fishing. Burbot are vulnerable to winter gillnet fishing and are targeted by commercial and subsistence fishers then. All fishers keep all of the fish they catch. |
|  | lenok (4) |  |
|  | burbot (3) |  |
|  | perch (1) |  |
|  | no response (0) |  |
| Is illegal fishing a significant problem in LHNP?* | rod fishing, no (5) | Most rod fishers are permitted and adhere to bag limits. Although 3 rangers say gillnet fishing is rare, 2 of these rangers report the confiscation of 60 gillnets in 3 years. |
|  | gillnet fishing, no (3) |  |
|  | gillnet fishing, yes (2) |  |
|  | no response (0) |  |
| How do rangers enforce fishing regulations?* | fine (4) | Rangers report fining illegal fishers based on the quantity of their catch and confiscate their gillnets. |
|  | confiscate nets (2) |  |
|  | no action necessary (1) |  |
|  | no response (0) |  |
| Have fish populations increased, decreased, or remained the same? | increased (2) | Most rangers report that fish populations have been decreasing and that lenok have become especially rare. |
|  | decreased (3) |  |
|  | remained the same (0) |  |
|  | no response (0) |  |
| Have fish body sizes increased, decreased, or remained the same? | increased (0) | The majority of rangers report that fish body size has remained the same. |
|  | decreased (1) |  |
|  | remained the same (3) |  |
|  | no response (1) |  |
| What should be done to protect fish?* | enforcement during spawn (2) |  |
|  | research, education, enforcement (1) |  |
|  | prevent upriver migration (1) |  |
|  | control cormorant population (1) |  |
|  | no response (0) |  |

* Multiple responses possible for these questions.
