# Supplementary figures and images for: A Mixed-Method Approach for Quantifying Illegal Fishing and Its Impact on an Endangered Fish Species
Source: PLoS One. 2015 Dec 1;10(12):e0143960. doi: 10.1371/journal.pone.0143960 (PMC4666464; doi:10.1371/journal.pone.0143960)

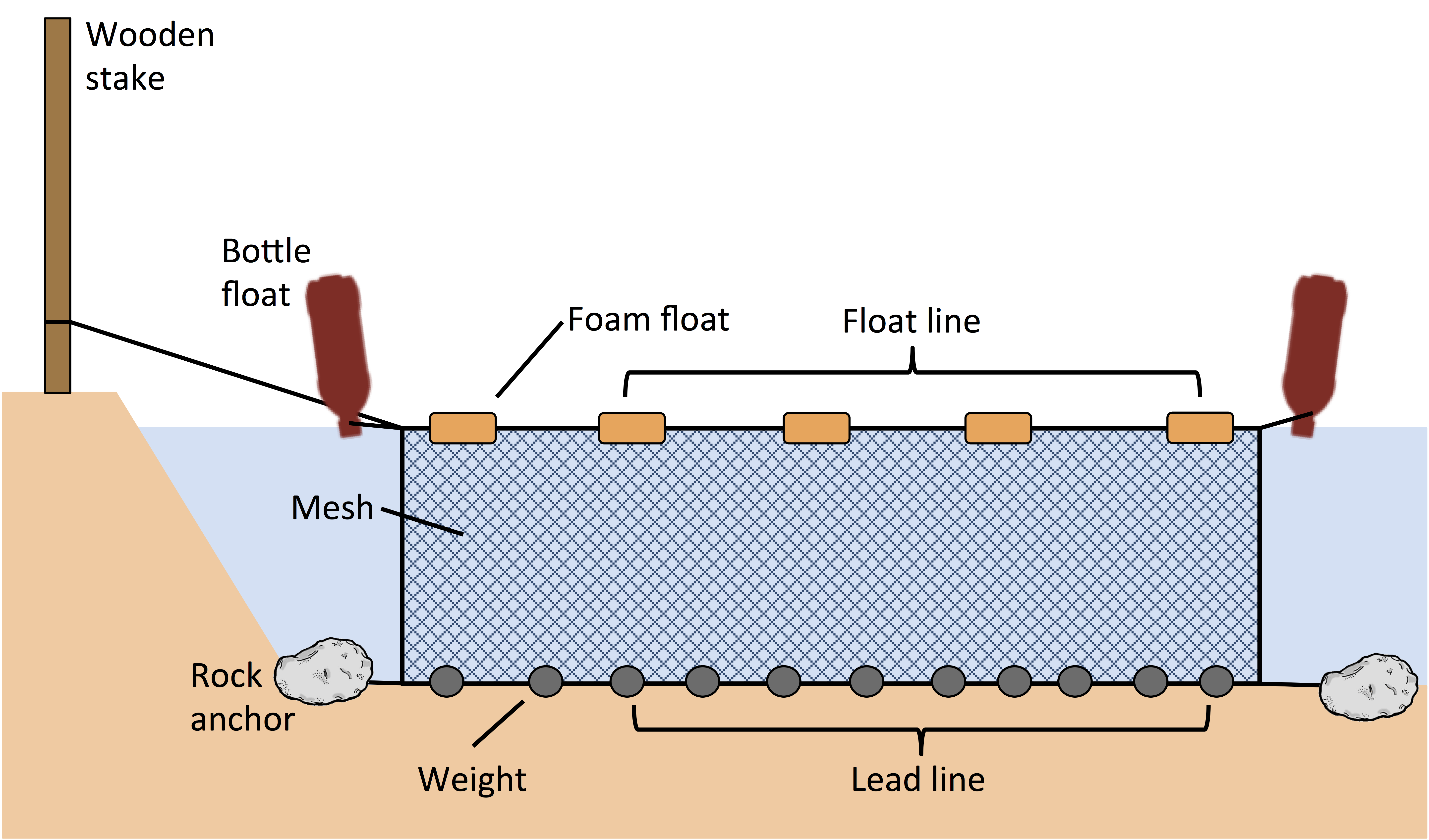

Supplement: S1 Fig — (PNG) [file pone.0143960.s006.png]

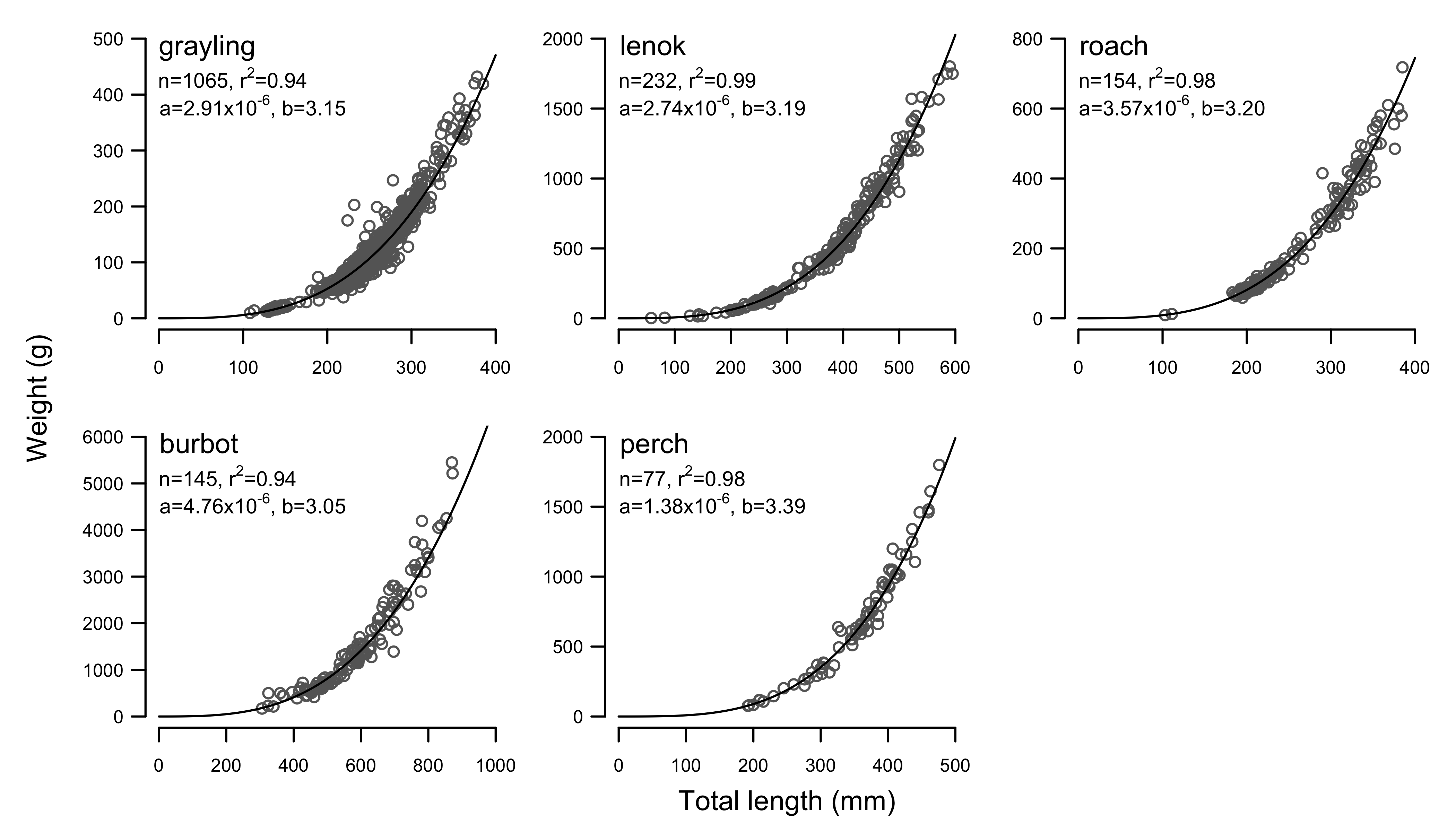

Supplement: S2 Fig — Note variable y-axis scales. (PNG) [file pone.0143960.s007.png]

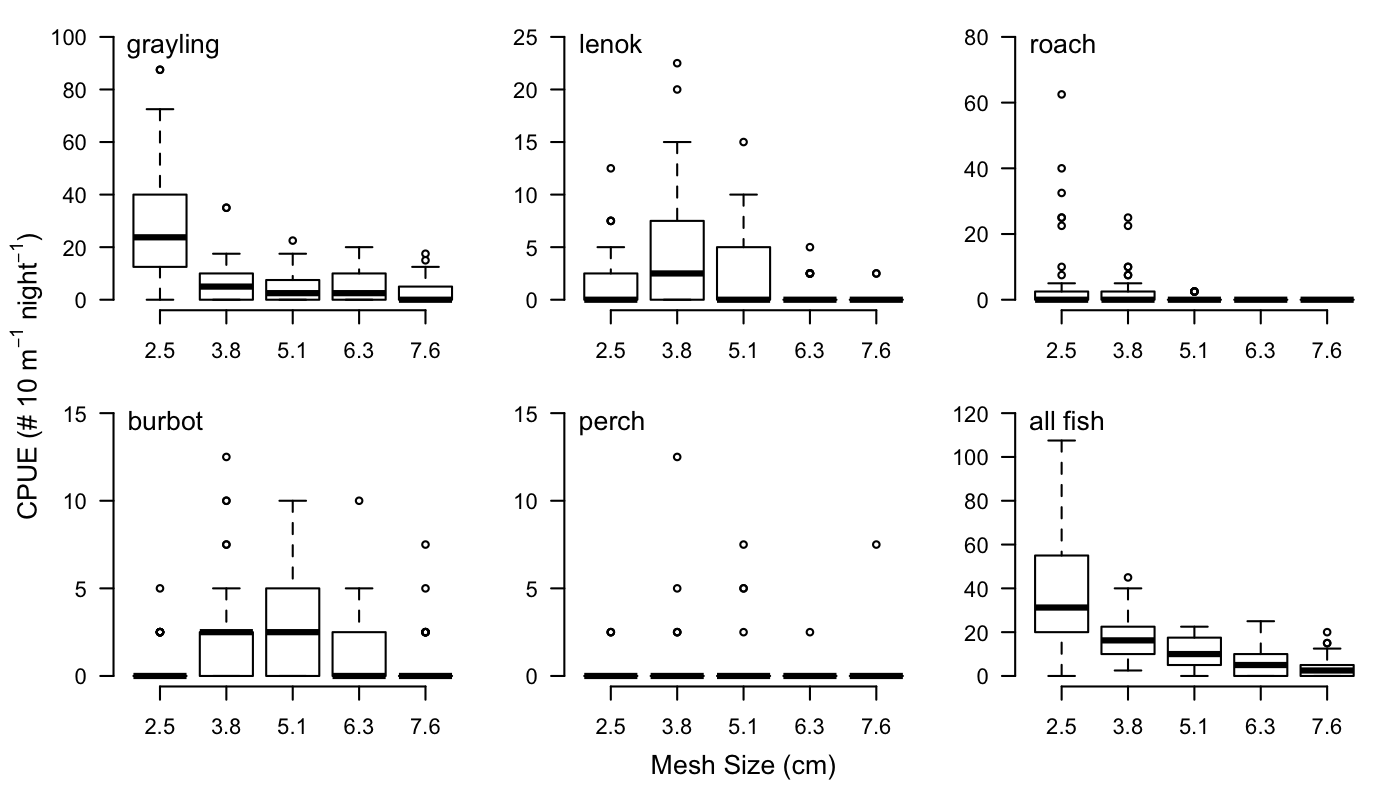

Supplement: S3 Fig — Data from the two 5-panel sequential mesh gillnets used at seven sites in 2009 and 2011–2013 (14 sets yr-1 x 4 yr = 56 sets total). Boxplots indicate median (heavy black line), interquartile range (IQR; box), 1.5 times the IQR (whiskers), and extreme values (open circles). Note variable y-axis scales. (PNG) [file pone.0143960.s008.png]

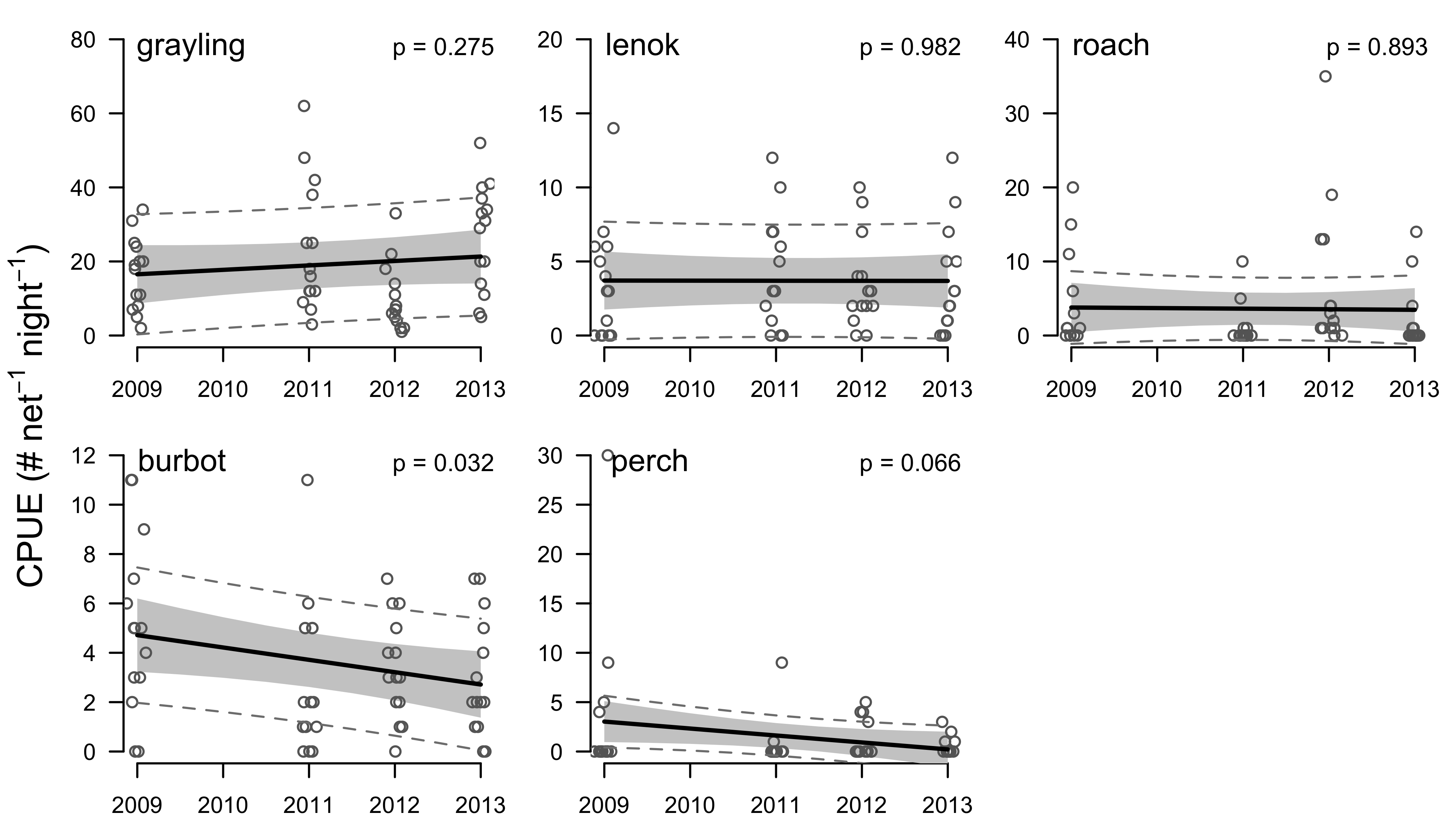

Supplement: S4 Fig — Points indicate the CPUE (# net-1 night-1) of each 5-panel sequential mesh gillnet set (2 nets site-1 x 7 sites yr-1 = 14 sets yr-1). Dark lines indicate linear mixed effects regressions fit to the catch data, gray shading indicates the confidence interval for each regression, and dashed lines indicate the prediction interval for the data. Points are jittered around year for display. P-values are indicated in the upper right corner of each panel. Note variable y-axis scales. (PNG) [file pone.0143960.s009.png]

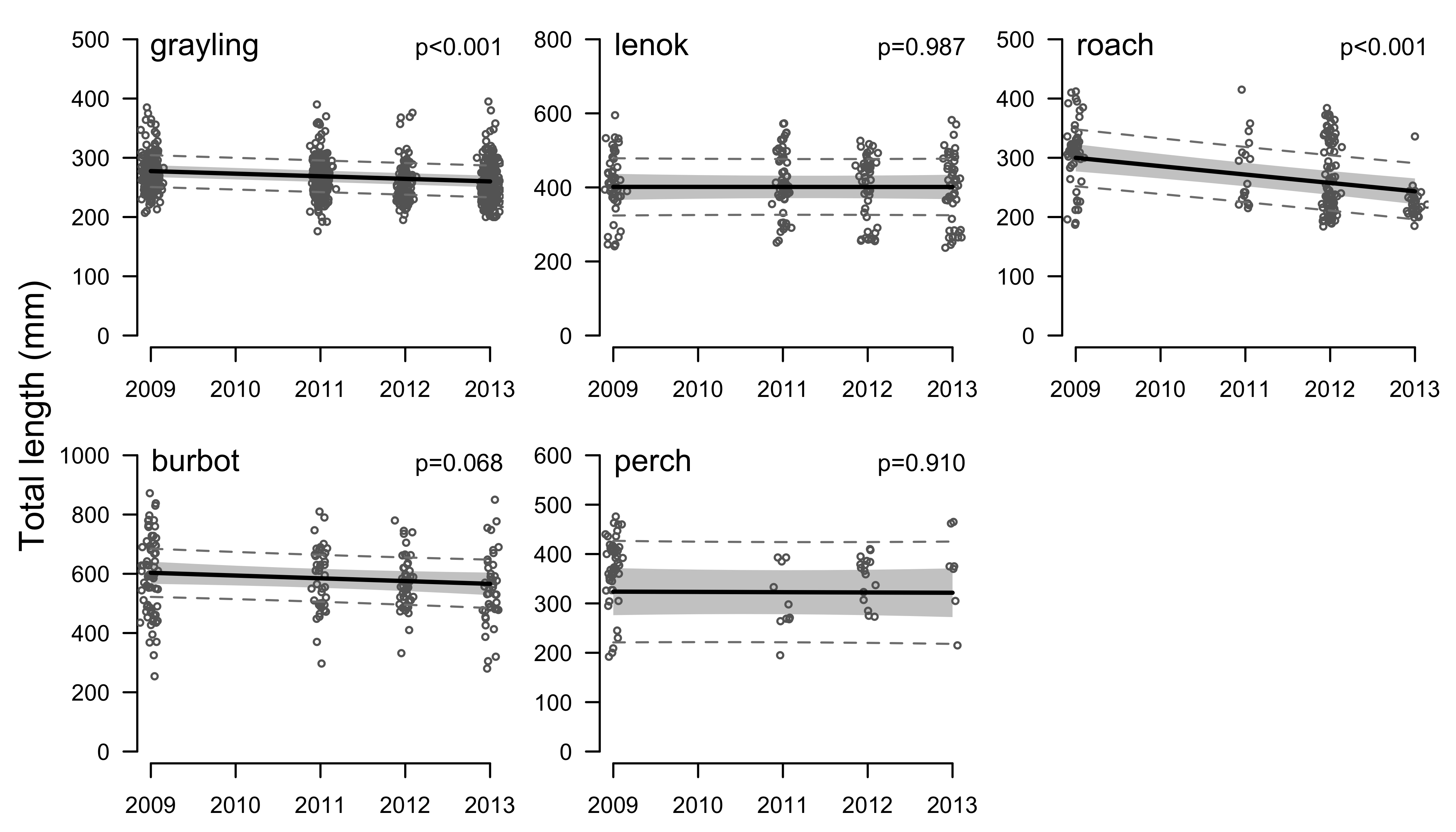

Supplement: S5 Fig — Points indicate the total length (mm) of every fish caught in gillnet sets that year (2 nets site-1 x 7 sites yr-1 = 14 sets yr-1). Dark lines indicate linear mixed effects regressions fit to the catch data, gray shading indicates the confidence interval for each regression, and dashed lines indicate the prediction interval for the data. P-values are indicated in the upper right corner of each panel. Points are jittered around year for display. Note variable y-axis scales. (PNG) [file pone.0143960.s010.png]

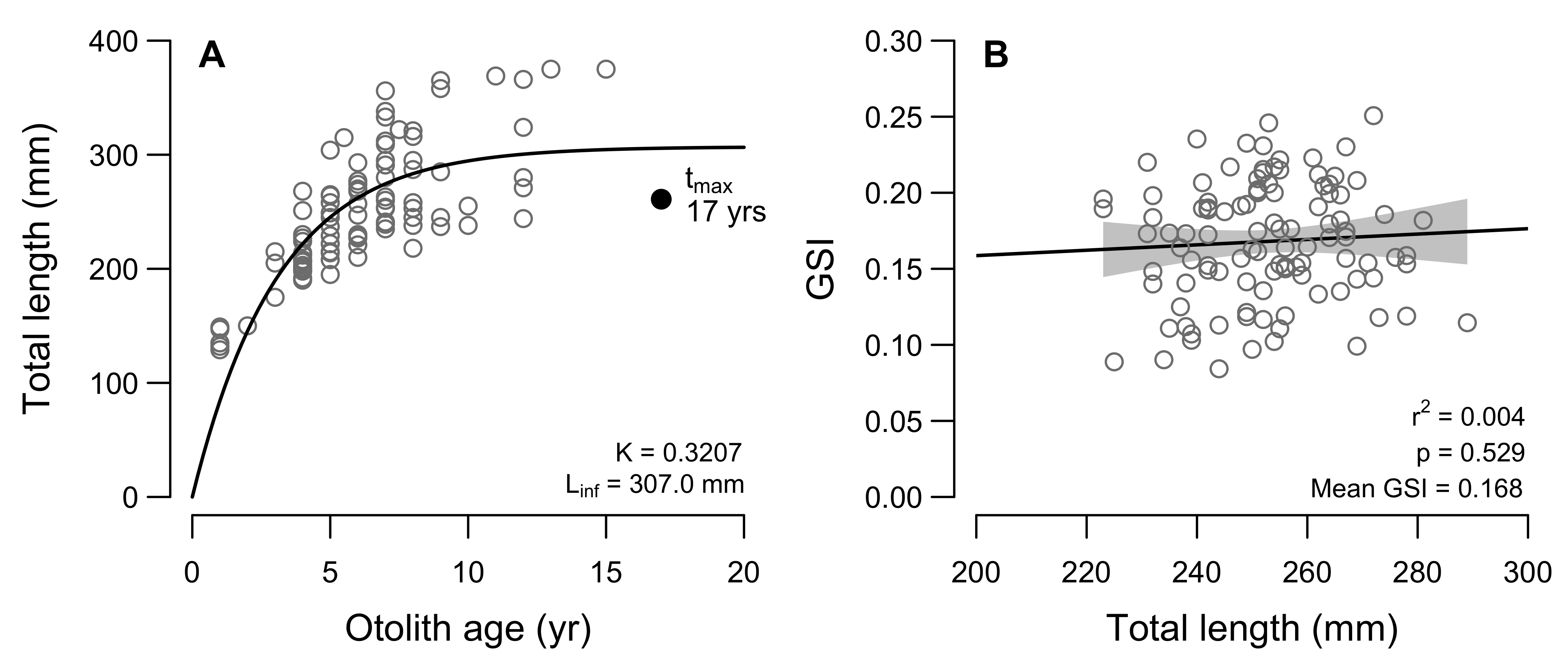

Supplement: S6 Fig — See Table 1for M estimation methods and results. In (A), L inf, K, and t max were estimated from aged otoliths and a von Bertalanffy growth model (black line) fit through the observed age-size relationship and origin (Tsogotsaikhan et al. in review). In (B), GSI was estimated as the mean gonadosomatic index (GSI) for all observed grayling (Jensen, unpublished data). In (B), the black line indicates a linear regression fit and the grey shading indicates the confidence interval for the regression. Life history characteristics are marked and labeled in both panels. (PNG) [file pone.0143960.s011.png]

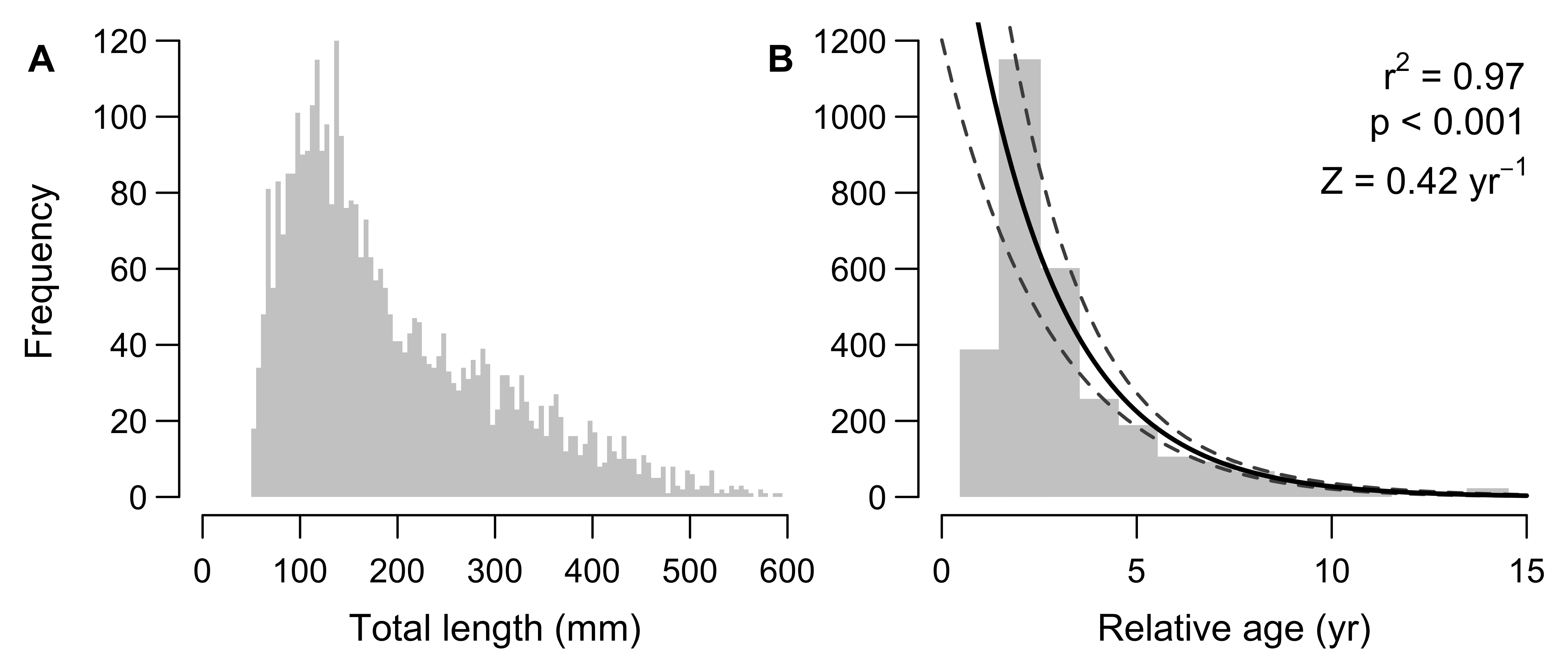

Supplement: S7 Fig — The length strucutre was observed in the Ahrenstorff et al. (2012) hydroacoustic surveys. In (B), the solid black line indicates a linear regression fit to the log-transformed trailing arm of the age structure. The dashed black lines indicate the confidence interval for the regression. Z is equal to the negative slope of the regression. (PNG) [file pone.0143960.s012.png]
