# Supplementary material for: A Mixed-Method Approach for Quantifying Illegal Fishing and Its Impact on an Endangered Fish Species
Source: PLoS One. 2015 Dec 1;10(12):e0143960. doi: 10.1371/journal.pone.0143960 (PMC4666464; doi:10.1371/journal.pone.0143960)
Supplement: S1 Appendix — (DOCX) [file pone.0143960.s013.docx]

**S1 Appendix.** Herder interview questionnaire.

**Opening questions**

1. Does your family eat fish?
2. How many times has your family eaten fish in the last month?
3. What types of fish does your family prefer to eat?
4. Does your family eat fish in all seasons?
5. How many people are in your family?
6. How many sheep, cows/yaks, goats, and horses does your family own?

**Personal fishing habits**

1. Does your family fish in Lake Hovsgol?
2. Does your family fish for recreation, food, or money?
   1. Do you keep all of the fish you catch? If not, which do you release?
3. How many times in the last month has your family fished?
4. For how many years has your family been fishing?
5. Who in your family fishes?
6. Where does your family go to fish?
7. How do you catch your fish?
   1. What type and how much net do you own?
   2. What type and how many rods do you own?
8. Where do you get your fishing equipment?
9. How many fish do you usually catch in a day of fishing?
10. What types of fish do you usually catch?

**Observed fishing habits**

1. Do you see other people fishing?
2. Are they mostly locals or foreigners?
3. Where do you see people fishing?
4. What type of gear do they use?
5. Do you know where you can buy this gear?
6. Do they fish for recreation, food, or money?

**Fish market questions**

1. Can you buy fish locally?
2. When and where can you buy fish?
3. What types and how much fish are usually available?
4. Does your family ever buy fish?
   1. What type of fish do you buy?
   2. How much fish and how frequently?
5. Are there any rules about fishing on Lake Hovsgol?

**Fish population questions**

1. Are fish more or less abundant than they used to be?
2. Are fish larger or smaller than they used to be?
3. What do you think should be done to protect the fish population?
