# Supplementary material for: A Mixed-Method Approach for Quantifying Illegal Fishing and Its Impact on an Endangered Fish Species
Source: PLoS One. 2015 Dec 1;10(12):e0143960. doi: 10.1371/journal.pone.0143960 (PMC4666464; doi:10.1371/journal.pone.0143960)
Supplement: S2 Appendix — (DOCX) [file pone.0143960.s014.docx]

**S2 Appendix.** Park ranger interview questionnaire.

**Opening Questions**

1. How long have you been a ranger?
2. What is your district?
3. How long have you worked in this district?
4. How many families live in your district?
5. How many families in your district fish?

**Observed Fishing Habits**

1. When do you see people fishing?
2. Which season is the most active for fishing?
3. How many people do you see fishing in a month?
4. Are they mostly local, visiting Mongolians, or foreigners?
5. Where do you see people fishing?
6. What type of fishing equipment do they use?
7. What type of fish do they catch?
8. What type of fish do they keep?
9. What type of fish do they release?
10. Do they fish for recreation, food, or money?
11. How many fish do they catch in a day of fishing?

**Law Enforcement Questions**

1. Are fishermen complying with the law?
2. What do you do when you see people fishing illegally?
3. Why do you give a fine sometimes and not other times?

**Fish Population Questions**

1. Are fish more or less abundant than they used to be?
2. Are fish larger or smaller than they used to be?
3. What do you think should be done to protect the fish population?
