# Supplementary material for: A Mixed-Method Approach for Quantifying Illegal Fishing and Its Impact on an Endangered Fish Species
Source: PLoS One. 2015 Dec 1;10(12):e0143960. doi: 10.1371/journal.pone.0143960 (PMC4666464; doi:10.1371/journal.pone.0143960)
Supplement: S3 Appendix — (DOCX) [file pone.0143960.s015.docx]

**S3 Appendix.** Total and natural mortality estimation.

*Total mortality: length-converted catch curve analysis*

We used a length-converted catch curve analysis (Pauly & Morgan 1987) to calculate the instantaneous total mortality (Z) rate where Z is the sum of the instantaneous natural (M) and fishing (F) mortality rates. First, we converted the length structure of Hovsgol grayling observed in the Ahrenstorff et al. (2012) hydroacoustic surveys into an age structure by (1) assigning observed fish to 5 cm length classes and (2) calculating the relative age of each fish based on the midpoint of its length class using the rearranged von Bertalanffy growth equation:

$$t_{i}=-log(1-\frac{L_{i}}{L_{inf}})/K$$

where *t_i_* is the mean age for the length class with midpoint *L_i_* and *L*_inf_ is 307.0 mm and *K* is 0.3206 yr^-1^ from Tsogotsaikhan et al. (*in review*). A linear regression was fit to the log-transformed trailing arm of the resulting age structure where the negative slope of the regression is equal to the instantaneous total mortality rate (Z).

*Natural mortality: life history invariant analysis*

Natural mortality rate (*M*) is one of the most important parameters in fisheries population dynamics and management but can be difficult and expensive to estimate directly. As a result, many authors have developed simpler, though necessarily less reliable, methods for indirectly estimating *M* from life history traits such as maximum age, Von Bertalanffy growth parameters, and maturity/reproductive characteristics. Kenchington (2014) provides the best review of such life history invariant methods to date. We evaluated the appropriateness of 31 life history invariant methods for estimating the natural mortality rate of Hovsgol grayling based on (1) the availability of required life history traits and (2) the performance and biases of each method (**S2 Table**). Ultimately, we decided to use three roughly independent estimators of natural mortality: Hoenig’s (Hoenig 1983), Pauly’s (Pauly 1980), and Gunderson’s estimators (Gunderson 1997). A recent paper by Then et al. (2014) suggests that Hoenig’s (t_max_-based) and Pauly’s estimators (growth-based) are the best in their respective classes and are independent of one another. Gunderson’s estimator is GSI-based (GSI=gonadosomatic index) and is therefore expected to be independent of the other estimators. Although Then et al. (2014) suggest that the Hoenig estimator performs best of the t_max_-and growth-based estimators and should be used alone, we consider three independent estimators to account for uncertainty in our life history trait estimates and uncertainty in the natural mortality estimates. See **S2 Table** for estimation methods considered, **Table 1** for estimation methods used, and **S6 Fig** for the life history traits required by the selected methods. See the section below for a description of the data sources and methods used to calculate these required life history traits.

*Natural mortality: life history trait data sources*

The Von Bertalanffy growth parameters (*L_inf_* and *K* with *t_0_* fixed at 0) and maximum age (*t_max_*) were determined from 93 aged otoliths in Tsogotsaikhan et al. (*in review*) (**S6 Fig A**). Thin sections through the core of the otoliths in the transverse plane were prepared and examined under a compound microscope (50x) using transmitted light. Alternating light and dark circuli, interpreted as annuli, were counted by two otolith readers. A von Bertalanffy growth model of length-at-age (*L_t_*):

$$L_{t}{=L}_{inf}\times\left( 1-e^{-K\left( t-t_{0} \right)} \right)$$

was fit to the resulting age and length data with *t_0_* fixed at 0. *L_inf_* (read “L-infinity”) represents the average maximum size or asymptotic length and *K* represents the rate at which *L_inf_* is approached. See Tsogotsaikhan et al. (*in review*) for more details.

The gonadosomatic index (*GSI*) was calculated for 106 grayling by dividing the wet ovary weight by the wet body weight (Jensen, unpublished data; **S6 Fig B**). Linear regression suggests that *GSI* does not vary with body length (r^2^=0.004, p=0.529) indicating that an average *GSI* value is representative of the entire grayling population.

We compared the indirect estimates of Hovsgol grayling natural mortality rate calculated here to direct estimates of natural mortality rate for Arctic grayling (*Thymallus arcticus*) from the literature to confirm realism (**S3 Table**).
