# Supplementary material for: A Mixed-Method Approach for Quantifying Illegal Fishing and Its Impact on an Endangered Fish Species
Source: PLoS One. 2015 Dec 1;10(12):e0143960. doi: 10.1371/journal.pone.0143960 (PMC4666464; doi:10.1371/journal.pone.0143960)
Supplement: S4 Appendix — (DOCX) [file pone.0143960.s016.docx]

**S4 Appendix.** Supplemental references.

**Mongolia fish and fisheries references**

Ahrenstorff TD, Jensen OP, Weidel BC, Mendsaikhan B, Hrabik TR. Abundance, spatial distribution, and diet of endangered Hovsgol grayling (*Thymallus nigrescens*). Environ Biol Fishes. 2012; 94(2): 465-476.

Dulmaa A. Fish and fisheries in Mongolia. In: Petr T, editor. Fish and fisheries at higher latitudes: Asia. FAO Fisheries Technical Paper 385. Rome, Italy: FAO; 1999.

Tsogtsaikhan P, Mendsaikhan B, Jargalmaa G, Ganzorig B, Weidel BC, Filosa CM, Free CM, Young T, Jensen OP. Comparison of age and growth of lenok, *Brachymystax lenok* (Pallas 1773), and grayling, *Thymallus* spp., in lentic and lotic habitats of Northern Mongolia. In review in J Appl Ichthyol.

**Life history invariant methods references**

Alagaraja K. Simple methods for estimation of parameters for assessing exploited fish stocks. Indian J Fish. 1984; 31(2): 177-208.

Alverson DL, Carney MJ. A graphic review of the growth and decay of population cohorts. ICES J Mar Sci. 1975; 36(2): 133-143.

Bayliff WH. Growth, mortality, and exploitation of the Engraulidae, with special reference to the anchoveta, *Cetengraulis mysticetus*, and the Colorado, *Anchoa naso*, in the eastern Pacific Ocean. Bulletin of the Inter-American Tropical Tuna Commission. 1967; 12(5): 365-432.

Beverton RJH, Holt SJ. A review of the lifespans and mortality rates of fish in nature, and their relation to growth and other physiological characteristics. In: Wolstenholme GEW, O’Connor M, editors. Ciba Foundation Symposium – the Lifespan of Animals (Colloquia on Ageing). Chichester, UK: John Wiley & Sons, Ltd; 1959. pp. 142–180.

Charnov EL, Berrigan D. Dimensionless numbers and life history evolution: age of maturity versus the adult lifespan. Evol Ecol. 1990; 4(3): 273-275.

Chen S, Watanabe S. Age dependence of natural mortality coefficient in fish population dynamics. Nippon Suisan Gakk. 1989; 55(2): 205-208.

Cubillos LA, Alarcón R, Brante A. Empirical estimates of natural mortality for the Chilean hake (*Merluccius gayi*): evaluation of precision. Fish Res. 1999; 42(1): 147-153.

Djabali F, Mehailia A, Koudil M, Brahmi B. A reassessment of equations for predicting natural mortality in Mediterranean teleosts. Naga ICLARM Q. 1994; 17(1): 33-34.

Frisk MG, Miller TJ, Fogarty MJ. Estimation and analysis of biological parameters in elasmobranch fishes: a comparative life history study. Can J Fish Aquat Sci. 2001; 58(5): 969-981.

Gislason H, Daan N, Rice JC, Pope JG. Size, growth, temperature and the natural mortality of marine fish. Fish Fish. 2010; 11(2): 149-158.

Griffiths D, Harrod C. Natural mortality, growth parameters, and environmental temperature in fishes revisited. Can J Fish Aquat Sci. 2007; 64(2): 249-255.

Groeneveld JC. Stock assessment, ecology and economics as criteria for choosing between trap and trawl fisheries for spiny lobster *Palinurus delagoae*. Fish Res. 2000; 48(2): 141-155.

Gunderson D. Trade-off between reproductive effort and adult survival in oviparous and viviparous fishes. Can J Fish Aquat Sci. 1997; 54(5): 990-998.

Hoenig JM. Empirical use of longevity data to estimate mortality-rates. Fish Bull. 1983; 82(1): 898-903.

Jennings S, Dulvy NK. Beverton and Holt’s insights into life history theory: influence, application and future use. In: Payne AI, Cotter AJR, Potter ECE, editors. Advances in Fisheries Science: 50 Years on From Beverton and Holt. Oxford: Blackwell; 2008. pp. 434-450.

Jensen AL. Beverton and Holt life history invariants result from optimal trade-off of reproduction and survival. Can J Fish Aquat Sci. 1996; 53(4): 820-822.

Jensen AL. Comparison of theoretical derivations, simple linear regressions, multiple linear regression and principal components for analysis of fish mortality, growth and environmental temperature data. Environmetrics. 2001; 12: 591–598.

Lorenzen K. The relationship between body weight and natural mortality in juvenile and adult fish: A comparison of natural ecosystems and aquaculture. J Fish Biol. 1996; 49(4): 627–647.

Kenchington TJ. Natural mortality estimators for information‐limited fisheries. Fish Fish. 2014; 15(4): 533-562.

Pauly D. On the interrelationships between natural mortality, growth parameters, and mean environmental temperature in 175 fish stocks. ICES J Mar Sci. 1980; 39(2): 175-192.

Pauly D, Morgan GR. Length-based methods in fisheries research. ICLARM Conference Proceedings 13. Manila, Philippines: International Center for Living Aquatic Resources Management; 1987.

Peterson I, Wroblewski JS. Mortality rate of fishes in the pelagic ecosystem. Can J Fish Aquat Sci. 1984; 41(7): 1117-1120.

Ralston S. Mortality rates of snappers and groupers. In: Polovina JJ, Ralston S, editors. Tropical Snappers and Groupers: Biology and Fisheries Management. Boulder, CO: Westview Press; 1987. pp. 375–404.

Richter VA, Efanov VN. On one of the approaches to estimation of natural mortality of fish populations. Kaliningrad, Russia: AtlantNIRO; 1977 (In Russian).

Roff DA. The evolution of life history parameters in teleosts. Can J Fish Aquat Sci*.* 1984; 41(6): 989-1000.

Sekharan KV. Estimates of the stocks of oil sardine and mackerel in the present fishing grounds off the west coast of India. Indian J Fish. 1975; 21(2): 177-182.

Tanaka S. Studies on the dynamics and the management of fish populations. Bull Tokai Reg Fish Res Lab. 1960; 28: 1-200 (In Japanese with English summary).

Ursin E. A mathematical model of some aspects of fish growth, respiration, and mortality. Can J Fish Aquat Sci. 1967; 24(11): 2355-2453.

Zhang CI, Megrey BA. A revised Alverson and Carney model for estimating the instantaneous rate of natural mortality. Trans Am Fish Soc. 2006; 135(3): 620-633.

**Arctic grayling life history references**

Buzby KM, Deegan LA. Inter-annual fidelity to summer feeding sites in Arctic grayling. Environ Biol Fishes. 2000; 59(3): 319-327.

Clark RA. Influence of stream flows and stock size on recruitment of Arctic grayling (*Thymallus arcticus*) in the Chena River, Alaska. Can J Fish Aquat Sci. 1992; 49(5): 1027-1034.

Clark RA. Stock assessment of Arctic grayling in Fielding Lake during 1991 and 1992. Fisheries Data Series No 93-2. Anchorage, AK: AK Department of Fish and Game; 1993.

Clark RA. Stock status and rehabilitation of Chena River Arctic grayling during 1994. Fisheries Data Series No 95-8. Anchorage, AK: AK Department of Fish and Game; 1995.

Fleming DF. Stock assessment of Arctic grayling in Piledriver Slough during 1994. Fisheries Data Series No 95-15. Anchorage, AK: AK Department of Fish and Game; 1995.
